# Microarray-Based Analysis of Methylation of 1st Trimester Trisomic Placentas from Down Syndrome, Edwards Syndrome and Patau Syndrome

**DOI:** 10.1371/journal.pone.0160319

**Published:** 2016-08-04

**Authors:** Lotte Hatt, Mads M. Aagaard, Cathrine Bach, Jesper Graakjaer, Steffen Sommer, Inge E. Agerholm, Steen Kølvraa, Anders Bojesen

**Affiliations:** 1 Department of Clinical Genetics, Vejle Hospital, Kabbeltoft 25, 7100 Vejle, Denmark; 2 Department of Gynecology and Obstetrics, Horsens Hospital, 8700 Horsens, Denmark; 3 Institute of Regional Health services research, University of Southern Denmark, 5000 Odense, Denmark; Hospital Authority, CHINA

## Abstract

Methylation-based non-invasive prenatal testing of fetal aneuploidies is an alternative method that could possibly improve fetal aneuploidy diagnosis, especially for trisomy 13(T13) and trisomy 18(T18). Our aim was to study the methylation landscape in placenta DNA from trisomy 13, 18 and 21 pregnancies in an attempt to find trisomy–specific methylation differences better suited for non-invasive prenatal diagnosis. We have conducted high-resolution methylation specific bead chip microarray analyses assessing more than 450,000 CpGs analyzing placentas from 12 T21 pregnancies, 12 T18 pregnancies and 6 T13 pregnancies. We have compared the methylation landscape of the trisomic placentas to the methylation landscape from normal placental DNA and to maternal blood cell DNA. Comparing trisomic placentas to normal placentas we identified 217 and 219 differentially methylated CpGs for CVS T18 and CVS T13, respectively (delta β>0.2, FDR<0.05), but only three differentially methylated CpGs for T21. However, the methylation differences was only modest (delta β<0.4), making them less suitable as diagnostic markers. Gene ontology enrichment analysis revealed that the gene set connected to theT18 differentially methylated CpGs was highly enriched for GO terms related to”DNA binding” and “transcription factor binding” coupled to the RNA polymerase II transcription. In the gene set connected to the T13 differentially methylated CpGs we found no significant enrichments.

## Introduction

Achieving non-invasive prenatal diagnosis of fetal cases of the common chromosome aneuploidies based on circulating free fetal DNA (cffDNA) in maternal plasma has for more than a decade been the goal of many research groups. The overall strategy for this research was to achieve sufficiently precise quantitation of e.g. chromosome 21 DNA fragments compared to fragments from a reference chromosome, so that a significant rise in chromosome 21 DNA fragments in maternal plasma could be demonstrated in pregnancies carrying a fetus with trisomy 21 (T21).

One approach to accomplish this is to measure circulating free DNA (cfDNA) in maternal blood with sufficient precision to demonstrate a significantly increased amount of e.g. chromosome 21 derived fragments, even when measuring the substantial background of maternal DNA fragments. This strategy has turned out to be successful for T21 when the quantitation is performed by Next generation sequencing (NGS). However, even though NGS has proven effective for T21 testing there are still problems with reliable demonstration of trisomy 13 (T13) and trisomy 18 (T18) with this approach[1]. Furthermore, the ability to detect increased chromosome dosage is very dependent on cffDNA fraction resulting in a minor fraction of non-reportable results.

An alternative approach to improve the method could be to obtain fetal DNA specificity by preferentially targeting fetal DNA sequences in the quantitative analysis. Thereby, likely lowering the limitation coming from the sensibility to low cffDNA fraction. This has been tried by several groups utilizing features such as DNA fragment length or epigenetic signatures, to distinguish the fetally derived DNA from the huge excess of maternal DNA [2–6]. In this context, we have in a previous study, using full genome methylation arrays, compared the methylation status of DNA from 12 placenta samples to DNA from 10 maternal blood samples, all from pregnancies in the first trimester [7]. We identified highly placenta specific epigenetic markers located on chromosome 13, 18, and 21 as well as highly placenta specific epigenetic markers in the regions of several microdeletion syndromes.

An alternative way to improve the discrimination between fetal and maternal cfDNA for non-invasive prenatal testing (NIPT) of the fetal aneuploidies would be to define fetal markers that are not only placenta specific but specific for trisomic placental DNA, preferable in the form of methylation differences. In agreement with this theory, two recent studies have looked into general epigenetic patterns in placental DNA from T21 cases and observed a general hypermethylation in T21 placentas compared to normal placentas [8,9]. We therefore extended our previous study by investigating the methylation landscape in placental DNA from the three common aneuploidies T21, T13, and T18 and compared it to the methylation landscape in DNA from normal placentas and from maternal blood cell (MBC) DNA in an attempt to demonstrate possible methylation differences better suited for NIPT especially for T13 and T18.

We have in addition looked into possible biological relevance of sites that differed in methylation between DNA from normal and trisomic placenta samples, firstly by characterizing the location of such sites within or between CpG islands and secondly by looking at the biological function of genes closest to the candidate sites using gene ontology.

## Materials and Methods

### Clinical samples

All the samples for the microarray study were sampled from 1^st^ trimester pregnant women, who underwent chorionic villus sampling (CVS) due to increased risk of T21, T18 or T13, estimated by a combination of the nuchal translucency testing and the double test (measuring the plasma protein markers; Pregnancy Associated Plasma Protein A (PAPP-A) and Chorionic Gonadotropin Beta (free β-hCG). We used 12 CVS samples from T21 pregnancies, 12 CVS samples from T18 pregnancies and 6 CVS samples from T13 pregnancies, all verified by chromosome analysis on CVS samples. We further used the data from our recent study [7] where we analyzed 12 CVS samples from normal pregnancies and 10 blood samples, from 1^st^ trimester pregnant women with a normal fetus, judged by a normal karyotype on CVS. An overview of all the samples including gestational age, maternal age and year of sampling can be viewed in the supporting information, S1 Table.

The project was approved by The Regional Committee on Health Research Ethics and The Regional Scientific Ethical Committees for Southern Denmark (Project no: S-20120042). The material used was excess DNA from routine investigation, stored at the biobank at the Clinical Genetic Department at Vejle Hospital. The samples were anonymized and de-identified prior to analysis. The institutional board at the Department of Clinical Genetics and The Regional Scientific Ethical Committees for Southern Denmark therefore waived the need for written informed consent for this study.

Processing of samples was done under identical conditions, however not all at the same time. The samples were not blinded.

### DNA extraction and quantification

#### Blood samples

DNA from blood samples were extracted using a standard salt extraction method as described in our previous study [7]

#### CVS samples

DNA from CVS samples was extracted using a QIAamp DNA Mini kit from Qiagen (QIAGEN Inc., Valencia, CA, USA) according to standard protocol provided by Qiagen. The samples were analyzed after separation of maternal decidua and without cell culture.

### DNA Quantification

DNA samples were quantified with a Nanodrop ND-1000 (Nanodrop Technologies, Wilmington, DE, USA)

### DNA methylation analysis–Infinium microarray analysis

The Illumina Infinium HumanMethylation450 Beadchip Kit (Illumina Inc., San Diego, CA, USA) was utilized for generation of methylation data for all samples. The analysis was done according to standard protocol provided by Illumina. Bisulfite conversion was done using a Zymo Research EZ DNA methylation kit (Zymo Research, Irvine, CA, USA). Beadchips were scanned with an Illumina HiScanSQ scanner using standard settings. Initial quality control, background subtraction and raw data normalization were done using the standard algorithms provided in Illumina Genome studio Methylation module v1.0. Methylation levels are quantified using β-values, as recommended by Illumina. Briefly, the β-value for each interrogated CpG site represents the fraction of methylated versus non-methylated probes, and consequently, the β-value ranges from 0 (unmethylated) to 1 (fully methylated). All analysis and statistical testing was performed on β-values.

### Data quality

The Infinium arrays include several control probes for determining data quality, including bisulphite conversion controls. Diagnostic plots of all control probes were visually inspected in the Genome studio software for the approval of each of our arrays. We have added the quality control plots in supporting information(S2 Fig).

Furthermore, 4 samples were analyzed in duplicates on different bead chip arrays. These replicates were analyzed to ensure reproducible data. Methylation data from the different beadchip showed very strong correlation between replicates (r>0.99). Validation data for the reproducibility of the 4 replicates can be viewed in S3–S5 Figs and S3 Table.

In addition, the microarray beadchip encompasses 65 probes for highly polymorphic single nucleotide polymorphisms (SNPs). When comparing the 65 polymorphic probes (correspond to genotype) between replicates, the status for each of these 65 sites was highly comparable between replicates (Spearman rho for each pair of replicates; 0.96 to 0.98). β-value of 0.0, 0.5, and 1.0 showed clear distinct patterns of homozygous or heterozygous methylation(Scatterplot of the correlation can be viewed in S6 Fig)

### Bioinformatics

Prefiltering and analysis of identified differentially methylated CpGs (DMCs) was performed as previously described [7], for all methylation profiles. In short, differences in methylation status between sample groups (e.g. CNOR vs T13) were evaluated for each CpG-site using the non-parametric Wilcoxon signed-rank test. The P-values were subsequently adjusted for multiple hypotheses testing using Benjamini-Hochberg correction. The resulting false discovery rates (FDRs) were used in combination with a Δβ-value cut-off (i.e. a minimum required difference in methylation level) between sample group means to define differentially methylated CpGs (DMCs). The addition of a Δβ-value cutoff in our definition of DMCs ensures that the observed methylation differences between sample groups (e.g. between the CNOR samples and the T13 samples), for a given CpG, is of a sufficient magnitude to be considered of biological relevance. The full dataset has been deposited in the gene Expression Omnibus (GEO) database (accession number GSE66210). In addition, the GOrilla web tool[10,11] was used for Gene Ontology(GO) analyses. The target and background gene lists were obtained by assigning each CpG to the nearest gene (RefSeq). Consequently, the GO analyses uses all genes represented (i.e. nearest gene) on the 450K array as background, and a small subset based on identified DMCs, as target list.

## Results

### General methylation landscape

**Fig 1:**
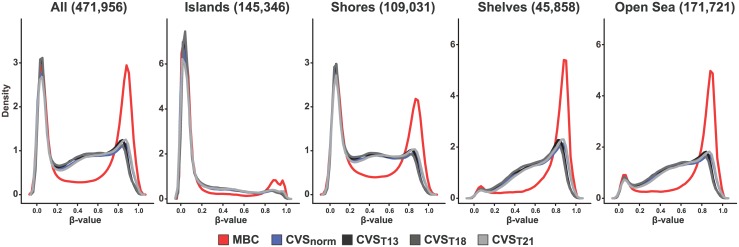
Density plot. Methylation level distribution for all sites and for CpG-islands, CpG shores (2 kb flanking CpG islands), CpG-shelves (2kb flanking CpG shores) and open sea (CpGs not mapping to islands, shores or shelves). X axis represents methylation level as mean β-values. Y axis represents relative density. Maternal blood cells (MBC) red line, CVS normal blue line, CVS T13 black line, CVS T18 dark grey line, T21 light grey line.

First the general methylation landscape in the different groups was described. Maternal white blood cells (MBCs) showed a very sharp and clear bimodal methylation distribution pattern (Fig 1). Thus, 36% of the CpGs for MBCs were hypomethylated (β-value<0.2) and 39% were hypermethylated (β-value>0.8, Table 1). The CVS samples also showed a somewhat bimodal distribution for the methylation of the CpGs, but the methylation pattern was partially shifted from hypermethylation to semimethylation. Hence, 35% of the CpG sites for normal diploid (CVS_NORM)_ were hypomethylated whereas only 16% were hypermethylated. The general methylation pattern observed for each of the trisomic CVS (T21, T18 and T13) closely resembled that of the normal diploid CVS (CVS_NORM_), although minor changes in the fraction of hypermethylated sites was observed compared to CVS_NORM_ (Table 1). Thus the fraction of hypermethylated sites for CVS_T21_ was slightly increased (17.81%), whereas it was decreased for CVS_T13_ and CVS_T18_ (13.96% and 12.64%, respectively). No major differences was observed in the methylation pattern for individual chromosomes except for chromosome X in MBC samples, which is probably caused by a substantial methylation of one of the X-chromosomes due to the X inactivation in females (S1 Fig).

**Table 1:** CpG methylation distribution.

	Hypomethylated β < 0.2		Hypermethylated β > 0.8	
	Number of sites	Percent	Number of sites	Percent
**MBC**	169392	35.89%	182223	38.61%
**CVS**_**NORM**_	164847	34.93%	77762	16.48%
**CVS T21**	158705	33.63%	84050	17.81%
**CVS T13**	163646	34.67%	65872	13.96%
**CVS T18**	164301	34.81%	59675	12.64%

The table lists the frequency of hypermethylated (mean β >0.8) and hypomethylation (mean β <0.2) CpG sites within each sample group. Maternal blood cells (MBC), chorionic villus samples(CVS) from normal (CNOR), CVS T21, CVS T18 and CVS T13.

Next we explored the methylation pattern of sites mapping to CpG-islands, CpG shores (2 kb flanking CpG islands), CpG-shelves (2kb flanking CpG shores) and open sea (CpGs not mapping to islands, shores or shelves). In general, the methylation pattern of sites mapping to each of these four regions is remarkably different with islands generally hypomethylated, shores bimodal methylated and shelves and open sea hypermethylated. In addition, placental DNA is generally less hypermethylated (and concomitantly more semimethylated) for all four regions, compared to MBC DNA (Fig 1).

### Differences in CpG methylation between DNA from MBCs, normal CVS samples and trisomic CVS samples

Comparing the methylation profiles of MBC and CVS_NORM_ revealed a huge number of DMCs in agreement with the findings shown in Fig 1. When comparing CVS_NORM_ to placental DNA from the different trisomies, only 3 DMCs between CVS_NORM_ and CVS _T21_ was identified, whereas 217 and 219 DMCs was identified for CVS _T18_ and CVS _T13_, respectively (Table 2, FDR<0.05). However, the mean difference in methylation level (β value) for CVS _T18_ and CVS _T13_ DMCs was in the range 0.2 to ~0.4, suggesting that all of the identified DMCs represent smaller methylation changes between placental DNA from normal and trisomic fetuses. Surprisingly, only 6 DMC’s overlapped between CVS _T18_ and CVS _T13_.

**Table 2:** Differential methylated CpGs (DMC)s.

	*Differentially methylated CpGs (DMC)*			
*FDR*	MBC v CVS_NORM_	T21 v CVS_NORM_	T13 CVS_NORM_	T18 CVS_NORM_
***<0*.*05***	143951	3	219	217
***<0*.*01***	143717	0	0	113
***<0*.*001***	142788	0	0	27
***<0*.*0001***	139494	0	0	0

The table lists the number of DMCs that hold a delta β >0.2 at different False Discovery Rate FDR. First column compares Maternal blood cells (MBC) to normal CVS samples (CNOR). The following columns compare T21, 18, and 13 CVS samples to normal CVS, respectively.

**Fig 2:**
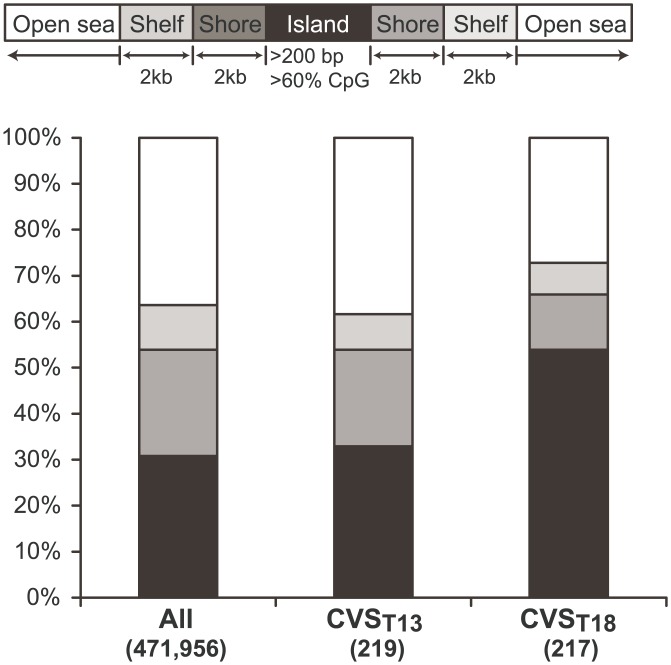
Stacked bar plot representation of the distribution of CpG sites. First column shows the distribution of all the 471,956 investigated CpG sites on the microarray split into CpG Islands (black), Shores (dark grey) Shelves (light grey) and open sea (White). Second and third columns show the distribution of sites that were significantly higher methylated in T13 and T18 compared to normal CVS samples.

Assessing the distribution of the DMCs in CVS _T18,_ we found an enrichment of the DMCs in CpG islands at the expense of shelves and open sea, when compared to the distribution of all CpG sites (Fig 2). DMCs in CVS _T13_ were not enriched in any location. We also investigated the distance of the CVS _T18_ DMCs to the transcription start site (TSS) for nearest gene, to see if the enrichment in CpG island was related to promotor regions, however we did not find any enrichment related to TSS.

### Gene ontology of T18 and T13 DMCs

To get an impression of the biological functions of genes closest to the T13 and T18 DMCs, Gene Ontology (GO) software was used. By associating each DMC to the nearest gene, the 219 CVS _T13_ and 217 CVS _T18_ DMCs were converted to gene-sets used for the GO-analyses. Only 3 DMCs was identified for T21, an inadequate number to establish a gene-set for a GO enrichment analysis. The CVS _T18_ gene-set was highly enriched for several GO terms related to”DNA binding” and “transcription factor binding” coupled to the RNA polymerase II transcription (Table 3a). Table 3b lists the 10 most significant enrichments of molecular functions and biological processes for the CVS _T18_ DMCs. In contrast, not a single GO term was enriched for the CVS _T13_ gene-set. The full list of enriched biological processes can be viewed in the supplementary material (S2 table).

**Table 3:** Gene Ontology(GO) analyses related to CVS T18 DMCs.

**Table 3 A**	**Molecular functions**				
**GO Term**	Description	FDR q-value	Enrichment	Genes within GO-term	Tri18 genes within GO-term
**GO:0043565**	sequence-specific DNA binding	2.50E-11	5.86	743	29
**GO:0003677**	DNA binding	2.14E-10	3.07	2346	48
**GO:0000981**	sequence-specific DNA binding RNA polymerase II transcription factor activity	1.27E-06	6.52	392	17
**GO:0003700**	sequence-specific DNA binding transcription factor activity	3.47E-06	3.77	1037	26
**GO:0001071**	nucleic acid binding transcription factor activity	2.83E-06	3.76	1038	26
**GO:0003676**	nucleic acid binding	4.86E-06	2.11	3770	53
**GO:0044212**	transcription regulatory region DNA binding	3.40E-05	4.93	518	17
**GO:0000975**	regulatory region DNA binding	3.61E-05	4.86	525	17
**GO:0001067**	regulatory region nucleic acid binding	3.21E-05	4.86	525	17
**GO:0001228**	RNA polymerase II transcription regulatory region sequence-specific DNA binding transcription factor activity involved in positive regulation of transcription	4.12E-05	8.22	201	11
**Table 3 B**	**Biological processes**				
**GO Term**	Description	FDR q-value	Enrichment	Genes within GO-term	Tri18 genes within GO-term
**GO:0045944**	positive regulation of transcription from RNA polymerase II promoter	1.37E-07	4.71	861	27
**GO:1903508**	positive regulation of nucleic acid-templated transcription	5.72E-07	3.72	1170	29
**GO:0019219**	regulation of nucleobase-containing compound metabolic process	6.15E-07	2.12	4105	58
**GO:0048518**	positive regulation of biological process	6.22E-07	2.03	4585	62
**GO:0045893**	positive regulation of transcription, DNA-templated	6.35E-07	3.72	1170	29
**GO:0045935**	positive regulation of nucleobase-containing compound metabolic process	6.51E-07	2.96	1880	37
**GO:0006357**	regulation of transcription from RNA polymerase II promoter	6.63E-07	3.33	1487	33
**GO:0006355**	regulation of transcription, DNA-templated	6.79E-07	2.34	3216	50
**GO:0051252**	regulation of RNA metabolic process	6.91E-07	2.29	3340	51
**GO:1903506**	regulation of nucleic acid-templated transcription	6.99E-07	2.32	3240	50
**GO:0051173**	positive regulation of nitrogen compound metabolic process	7.01E-07	2.91	1907	37

A: Gene functions related to terms within the group “Molecular functions”. B. Gene functions related to terms within the group “Biological processes”. The table shows the enrichment analysis for the genes associated to our DMCs in T18 samples. The total number of genes within the GO analysis is 17727. Our gene set related to the CVS T18 DMCs were 118. Each table shows the 10 GO terms with the lowest False Discovery Rate (FDR q value).

## Discussion

Using methylation array technology we have compared the methylation landscape between MBC DNA and placental DNA and furthermore between normal placental DNA and placental DNA from the three common trisomies, with the aim of exploring if these epigenetic differences could improve cffDNA-based non-invasive prenatal diagnosis (NIPD).

A clear difference in the overall methylation pattern between MBC DNA and all four types of placental DNA was observed. Compared to DNA from maternal blood, CVS samples are substantially less hyper-methylated (with a concomitant increased fraction of semi-methylated sites), corroborating recent observations from two studies [9,12].

With regards to the trisomic samples, we observed that the fraction of fully methylated sites were slightly increased in CVS _T21_ and decreased in CVS _T13_ and especially in CVS _T18_ compared to CVS_NORM_. Exploring this closer by looking at number of sites that were significantly different methylated in CVS _T21_, CVS _T13_ and CVS _T18_ DNA compared to CVS_NORM_ DNA we observed very few DMC’s in CVS _T21_ DNA and in contrast a substantial and very similar number of DMC’s for CVS _T13_ and CVS _T18_ DNA but with a very limited amount of overlap. The DMC’s in T18 were more robust–lowering the FDR (increasing level of significance) lead to clear differences between T18 and T13 (see Table 2). However, it should be remembered that the sample sizes were different since, we could only include six T13 samples due to the rarity of T13 pregnancies. We do not know the explicit reason as to why T13 and T18 have a higher number of DMCs compared to T21, however it does coincide with the more severe phenotypes for T13 and T18 in which the affected foetuses often die in the uterus or within the first few years of life as compared to T21 affected foetuses.

Subsequently Gene Ontology enrichment analysis showed for CVS _T13_ DMC’s that among the gene functions of the nearest genes, there was no significant overrepresentation of specific functions compared to the reference gene set. In contrast, among the functions of the nearest gene to the CVS _T18_ DMC’s, there was a highly significant overrepresentation of gene functions related to regulation of RNA polymerase II mediated transcription and sequence-specific DNA binding related to transcription factor activity. The smaller number of T13 samples does, however, increase the risk of false negative findings in the GO analysis, but in spite of this we find the difference in significant GO terms between CVS _T18_ and CVS _T13_ interesting. We have at present no explanation for this difference but as DNA methylation to some extent can be considered a weak proxy for gene expression it would be interesting to investigate if there are differences in gene expressions of genes involved in regulation of RNA polymerase II mediated transcription between CVS _T18_ and CVS _T13_ samples.

We are aware that caution should be taken in the interpretation of microarray based data from CVS samples, because the chorionic villi is a heterogeneous mixture of syncytiotrophoblastic-, cytotrophoblastic-, mesodermal- and fetal endothelial/vascular cells, and the proportion of the different cell populations in the biopsy could be a confounding factor. This is, however, to our knowledge the first study to date to investigate the methylation landscape in T13 and T18 placentas. The relatively large number of samples, especially for T13 and T18 placentas, minimizes the risk of random variability and therefore provides a more representative biological measure. To further limit the risk of confounding factors such as sex and gestational age we have used gestational–age-matched blood samples with an equal distribution of samples with male and female fetuses.

Regarding the circulating cell free DNA (cfDNA) we choose to use maternal white blood cells as a proxy, since cfDNA in plasma from non-pregnant individuals predominantly originates from hematopoietic cells [13]. Therefore, it is assumed that the maternally derived cfDNA in blood from pregnant women has the same origin. However, one study has suggested that for very obese women a substantial fraction of adipose apoptotic DNA could be released into the maternal systemic circulation [14]. Optimally maternal cfDNA should have been used. However, the very low cfDNA fraction in blood samples hinders its appliance for microarray analysis.

Only very few publications have until now looked at methylation in trisomic placental DNA. However, the observation of a small but significant increase in fraction of hypermethylated CpG sites in placental samples from T21 cases compared to samples of normal placenta, are in line with two recent publications showing a general hypermethylation across all chromosomes in fetal DNA from T21 placentas [8,9]. Furthermore Jin *et al*. found several of the epigenetic changes to be conserved across tissues and they proposed that a down regulation of a group of the *TET*-family genes known to play an important role in epigenetic regulation could be responsible for the overall hypermethylation seen in all chromosomes through decreased DNA demethylation[9,15]. However, we identified only three CVS _T21_ DMCs using the most lenient criteria (delta β>0.2 & FDR<0.05), suggesting that the methylation differences are either relatively small or not very consistent. Eckmann-scholz *et al*. found 464 DMC between normal and T21 CVS samples, with a delta β >0.2. They applied the same Illumina methylation arrays as in our study, however only assessing 27.000 CpGs and the number of T21 samples in their study was only 3[8].

Assessing the overall methylation landscape in relation to the distance to CpG islands, we found the most highly different methylated CpGs in T18 to be enriched in CpG islands and decreased in open sea and shores, when compared to the distribution of all the covered CpGs. However, when investigating the distribution of CVS _T18_ DMC in relation to transcription start sites (TSS), we did not find any enrichment of the DMCs in TSS vicinity, indicating that the CVS _T18_ DMCs are located at non-promotor associated CpG islands

We have in the present communication provide a detailed methylation analysis of samples from T13, T18 and T21 placentas and compare them to normal placenta tissues and maternal blood. We found a substantial number of significant CpG methylation differences in T13 and T18 placenta DNA compared to T21 placenta DNA. Our data suggests that the genes associated with CVS T18 DMCs are enriched for biological pathways/processes related to RNA polymerase II mediated gene expression. Our findings do not support the idea of using differences in the specific methylation imprint (or landscape) of T13, T18 or T21 as targets for NIPD.

## Supporting Information

S1 Fig Density plot of methylation level distribution for CpG-sites on different chromosomes. X axis represents methylation level as mean β-values. Y axis represents relative density. Maternal blood cells (MBC) red line, CVS normal blue line, CVS T13 black line, CVS T18 dark grey line, T121 light grey line.
(EPS)

S2 Fig Diagnostic quality control plots. The Illumina Infinium HumanMethylation450 Beadchip arrays include several control probes for determining data quality, including sample-independent controls: staining controls, extension controls, target removal controls and hybridization controls. Sample-dependent controls: Bisulphite conversion I controls, Bisulphite conversion II controls, Specificity I controls, and Specificity II controls, Nonpolymorphic controls and Negative controls. Diagnostic plots of all control probes, visualized by illumines genome studios software, are presented for each of the 3 beadchip arrays.
(ZIP)

S3 Fig Validation of DNA methylation data reproducibility. Four replicate samples, two maternal blood samples (MBC) and two CVS-samples, underwent independent bisulphite conversion and were analyzed on different beadchip arrays for the validation of DNA methylation data reproducibility. The figure shows hierarchical clustering and heatmap of correlation coefficients based on all 472K methylation sites. The four replicates MBC3, MBC6, CNOR3 and CNOR5 all shows the highest correlation between all samples.
(PDF)

S4 Fig Density scatter plots of the four replicates. All four replicates have a correlation coefficient > 0.99 for all methylation sites.
(JPG)

S5 Fig Reproducibility and completeness of bisulphite conversion of unmethylated sites. The figure shows the mean delta β values for all unmethylated sites (average β < 0.2) for the four replicates. Since the replicates underwent independent bisulphite conversion, complete bisulphite conversion of unmethylated sites should results in reproducible low beta values. A. Delta β values for all unmethylated sites for the two maternal blood cell (MBC) replicate samples. Sorted with decreasing delta β values. X axis represents the numbers of unmethylated sites. B. The distribution of the 100 highest delta β values for the MBC samples. C. Delta β values for all unmethylated sites for the two CVS replicate samples (CNOR). D. The distribution of the 100 highest delta β values for the CVS samples.
(PDF)

S6 Fig Scatterplot displaying data reproducibility of highly polymorphic single nucleotide polymorphisms (SNPs). The beadchip encompass 65 highly polymorphic single nucleotide polymorphisms (SNPs) The scatterplot displays the β-values for 65 polymorphic SNPs for the four replicate samples. Spearman correlation coefficient (ρ) for each pair of replicates is shown. Diagonal red line represents complete correlation (ρ = 1.00). Methylation levels (β-value) of 0,0, 0.5, and 1.0 shows clear distinct patterns of homozygous or heterozygous methylation.
(EPS)

S1 Table Overview of all samples used in the methylation analysis. The table lists samples, fetal gender, year of sampling, sample material, chromosome analysis, gestational age (weeks), and maternal age (in years).
(DOCX)

S2 Table Gene Ontology (GO) analyses related to CVS T18 DMCs. Gene functions related to terms within the group “Biological processes”. The table shows the enrichment analysis for the genes associated to our DMCs in T18 samples. The total number of genes within the GO analysis is 17727. Our gene set related to the CVS T18 DMCs were 118.
(XLS)

S3 Table Overview of the methylation differences between replicates. Numbers (and percentages) of sites with increasing delta β value thresholds. A All methylation sites. B Unmethylated Sites (average delta β < 0.2).
(PDF)
