# Supplementary figures and images for: Microarray-Based Analysis of Methylation of 1st Trimester Trisomic Placentas from Down Syndrome, Edwards Syndrome and Patau Syndrome
Source: PLoS One. 2016 Aug 4;11(8):e0160319. doi: 10.1371/journal.pone.0160319 (PMC4973974; doi:10.1371/journal.pone.0160319)

Quality control plot array 1

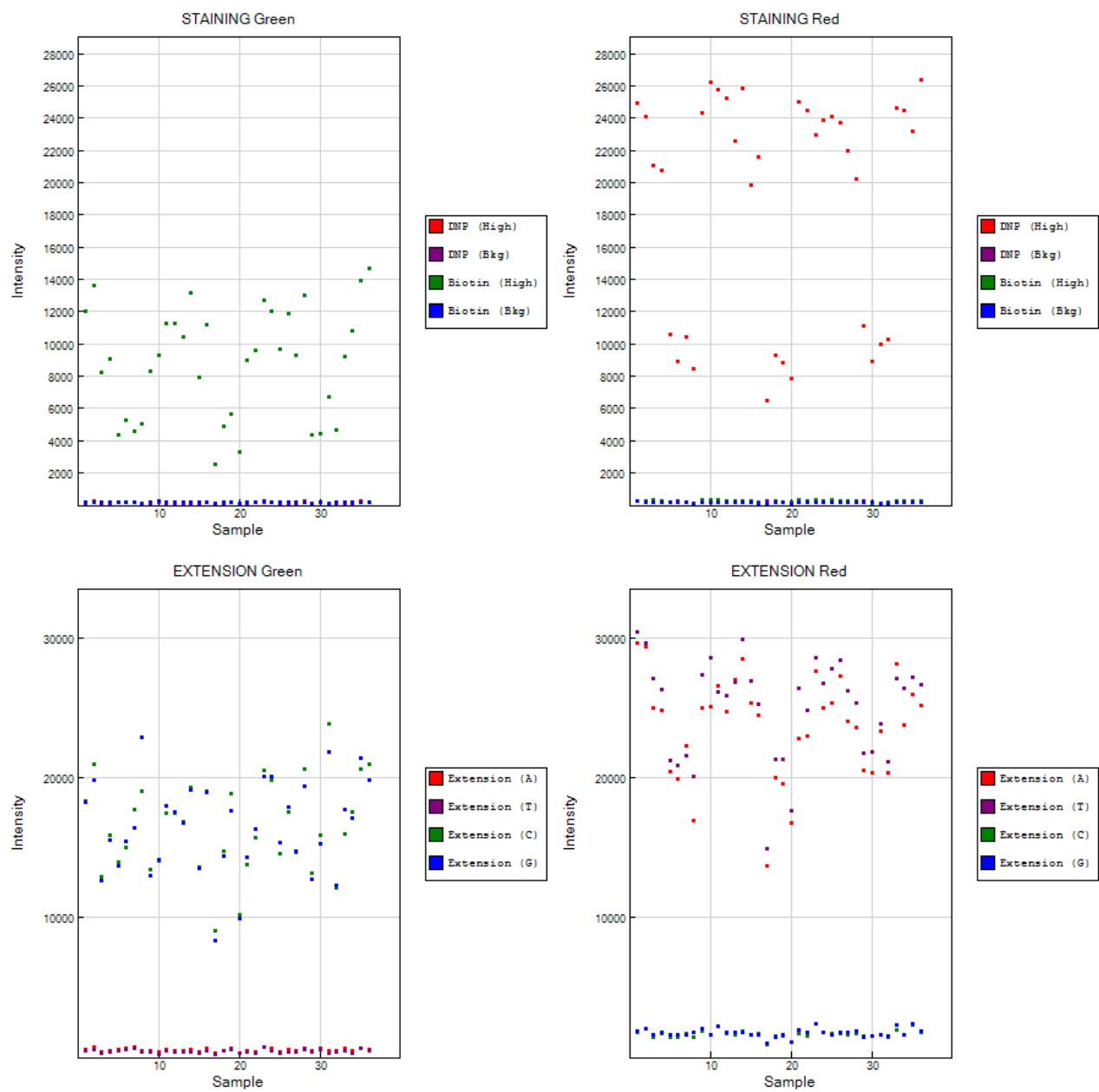

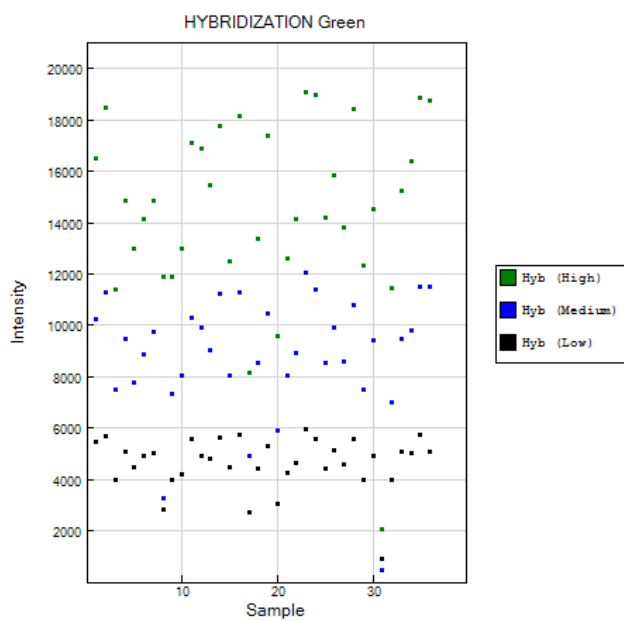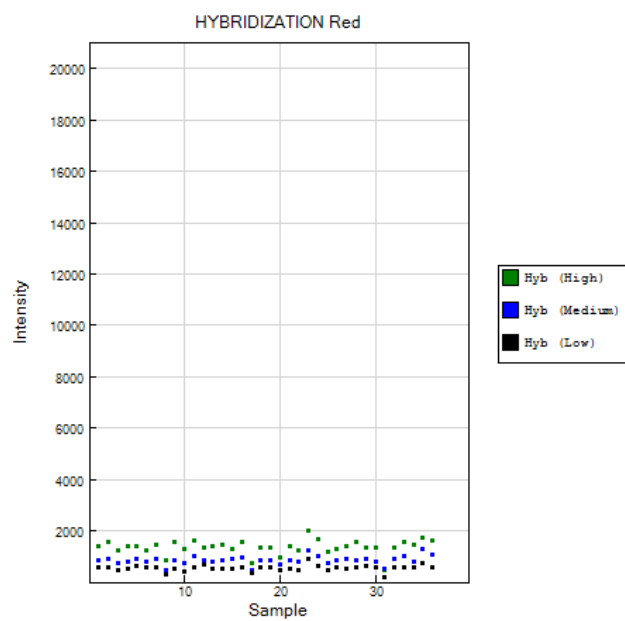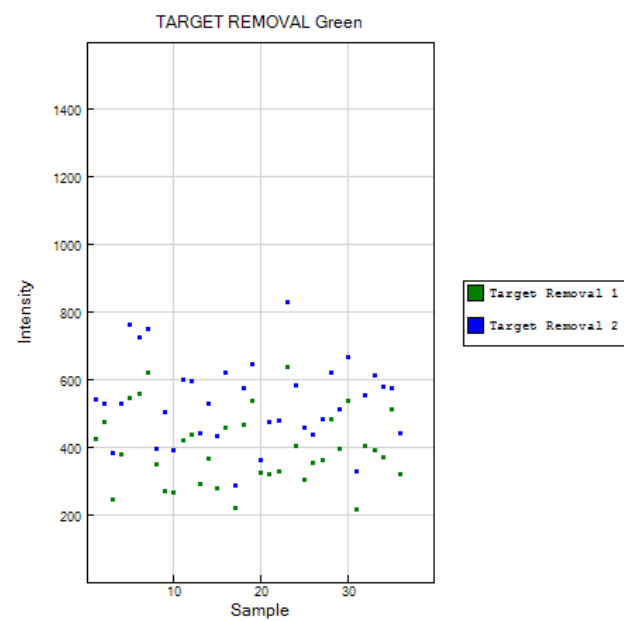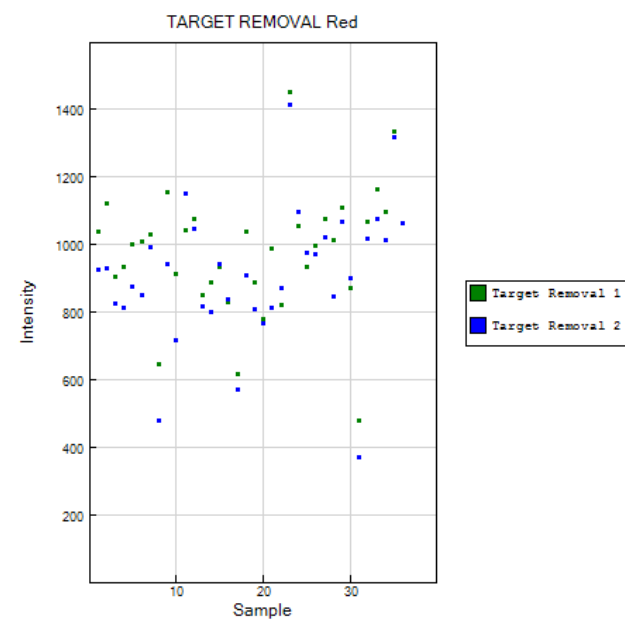

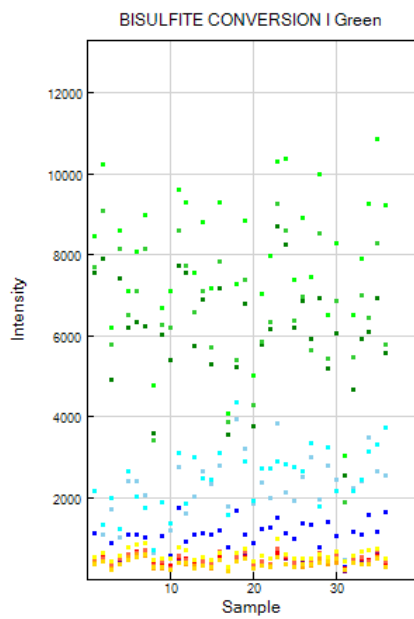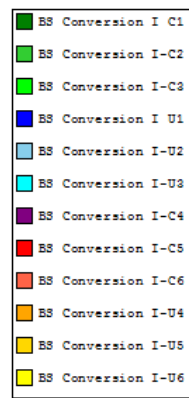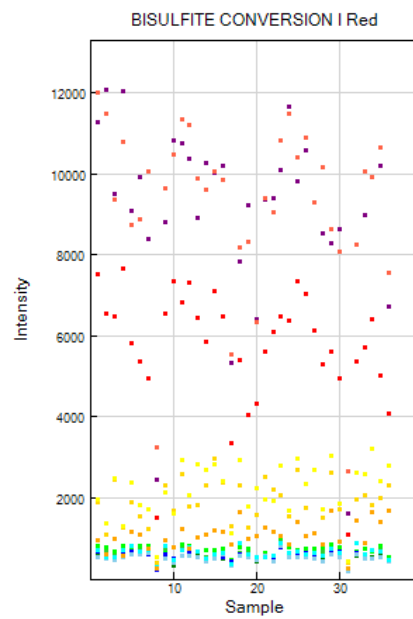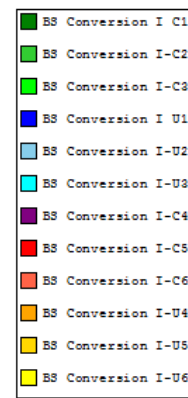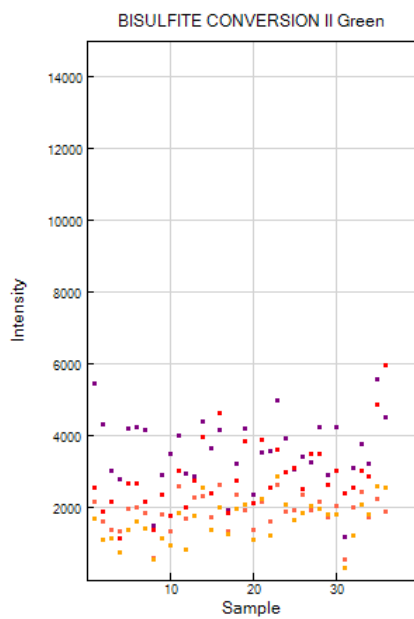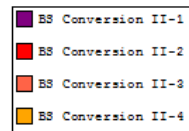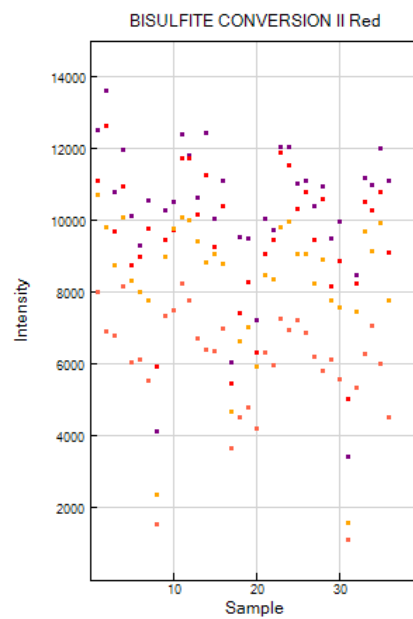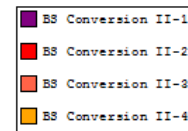

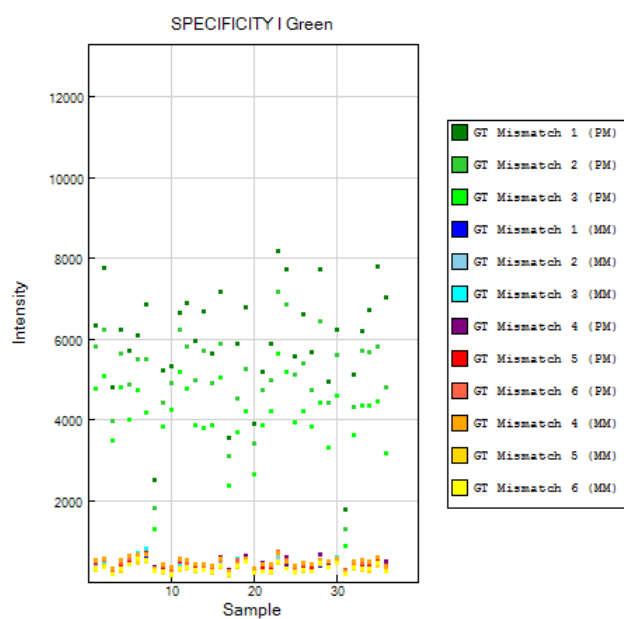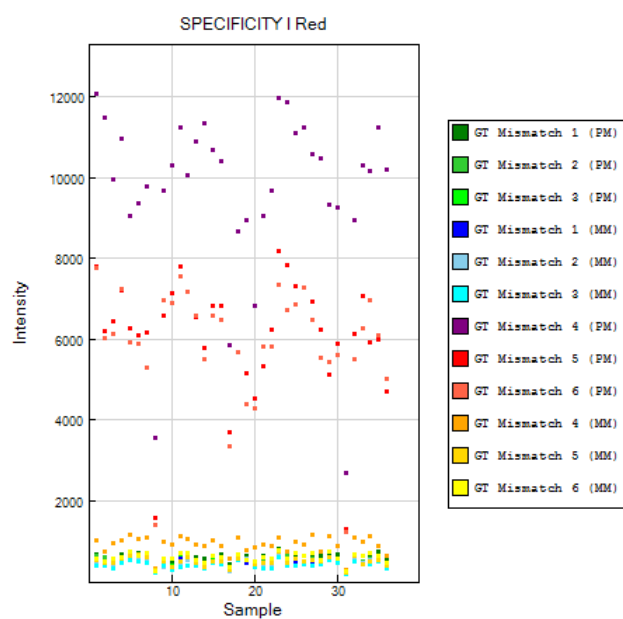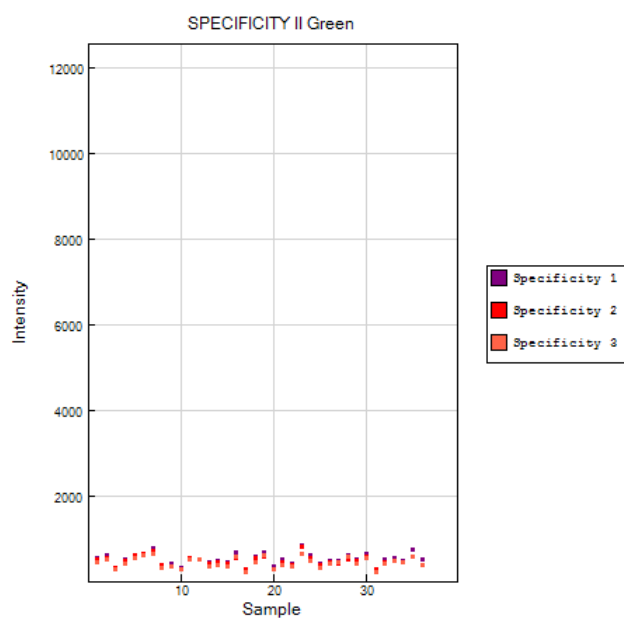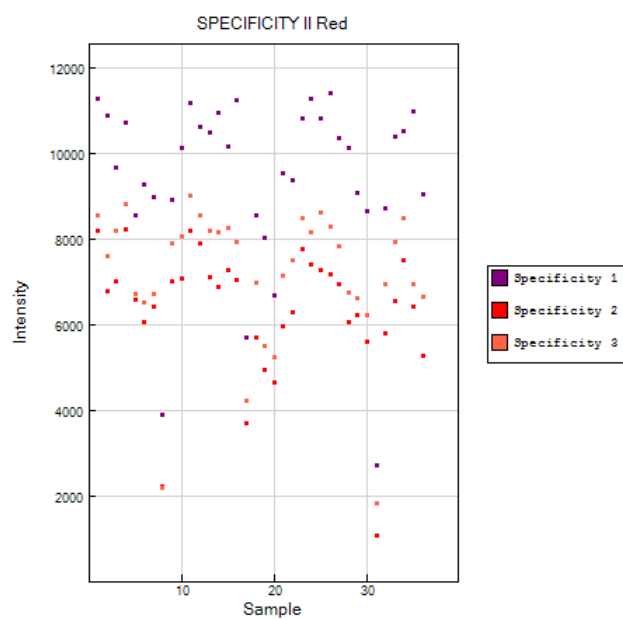

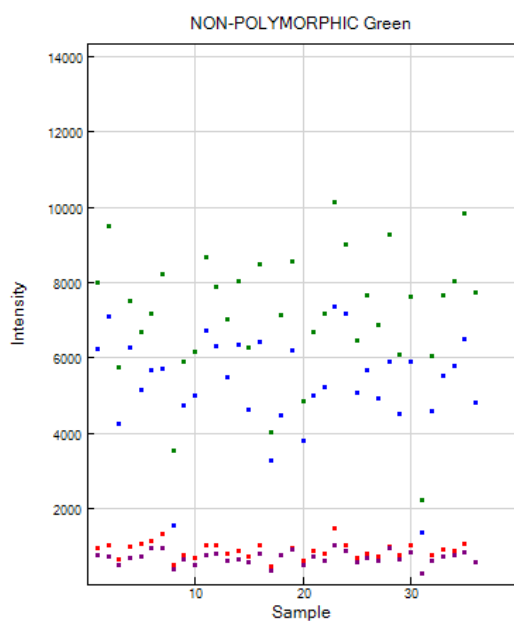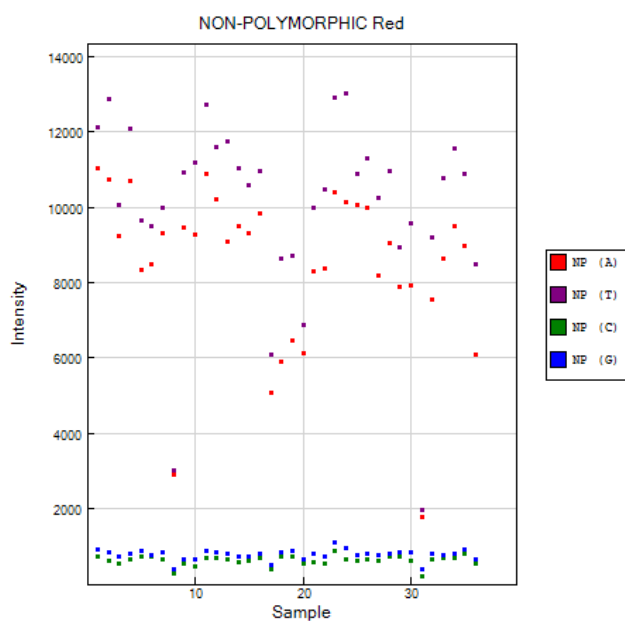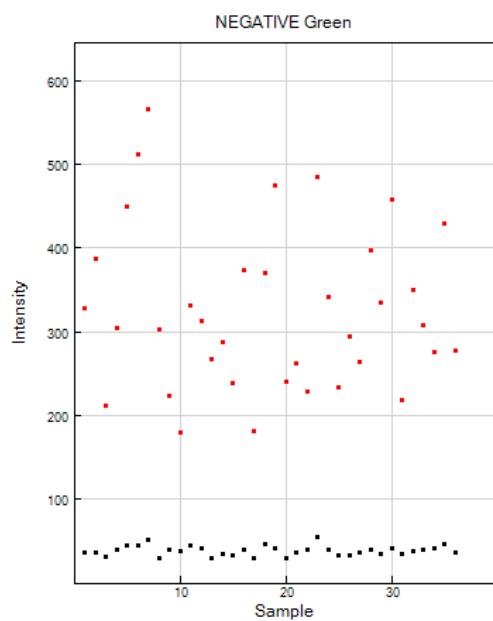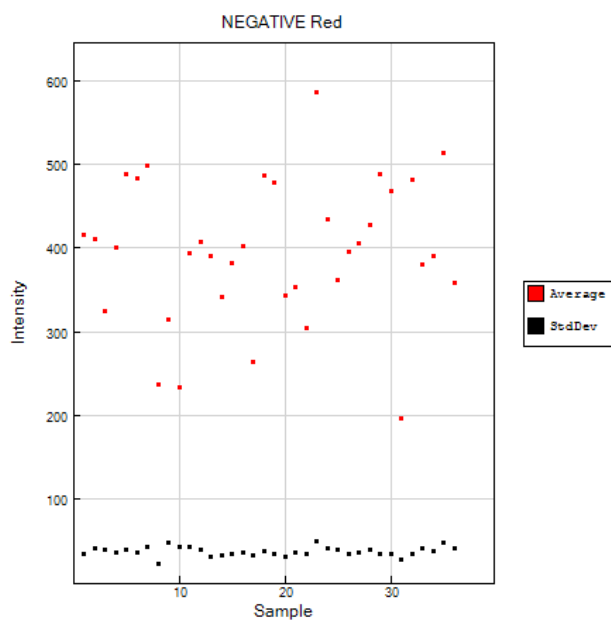

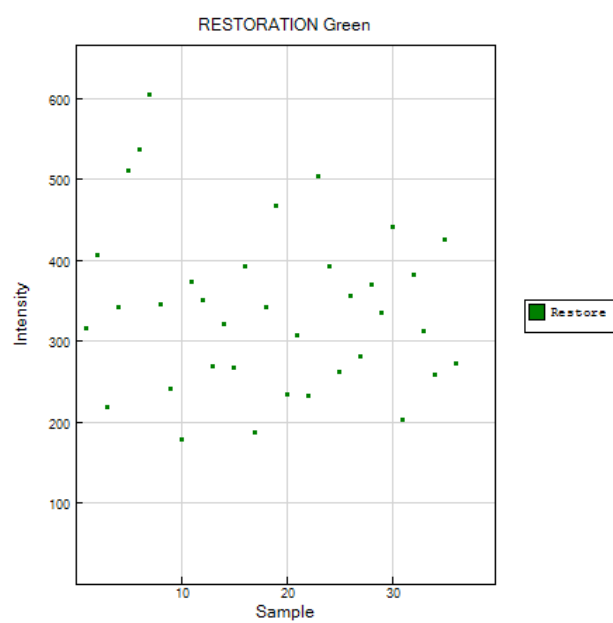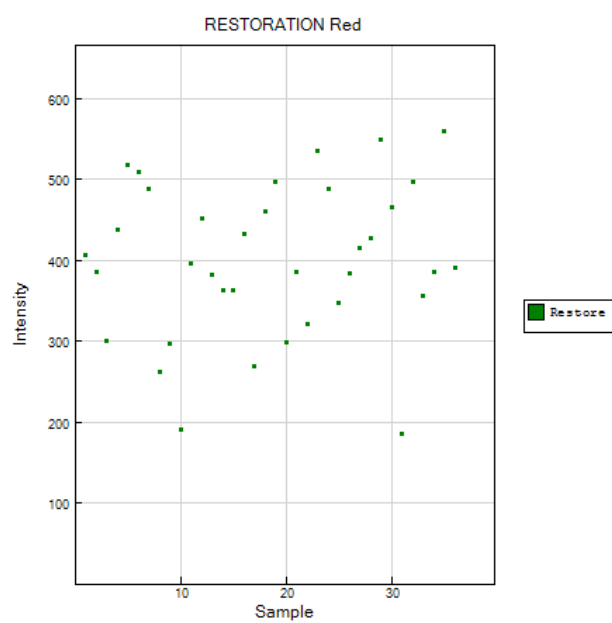

Supplement: S2 Fig — The Illumina Infinium HumanMethylation450 Beadchip arrays include several control probes for determining data quality, including sample-independent controls: staining controls, extension controls, target removal controls and hybridization controls. Sample-dependent controls: Bisulphite conversion I controls, Bisulphite conversion II controls, Specificity I controls, and Specificity II controls, Nonpolymorphic controls and Negative controls. Diagnostic plots of all control probes, visualized by illumines genome studios software, are presented for each of the 3 beadchip arrays. (ZIP) [file pone.0160319.s002.zip › Quality plots array 1.pdf]

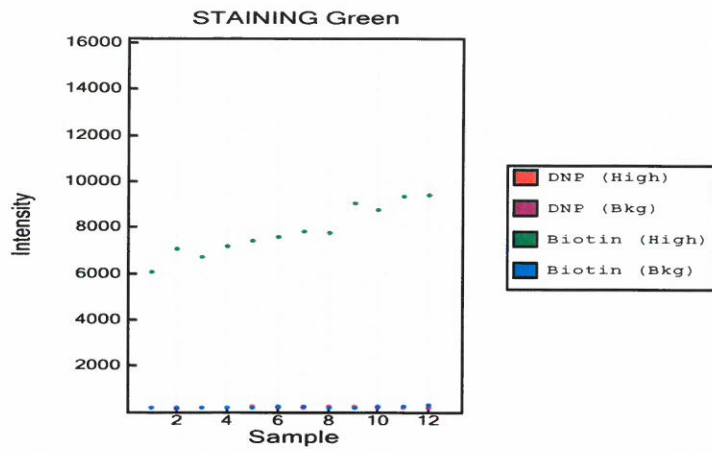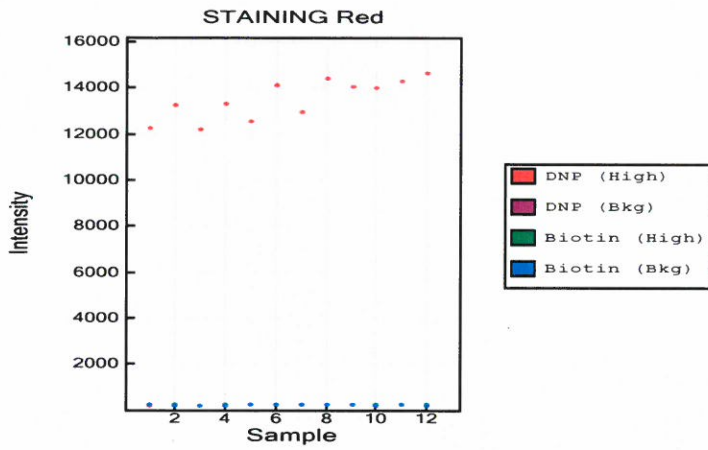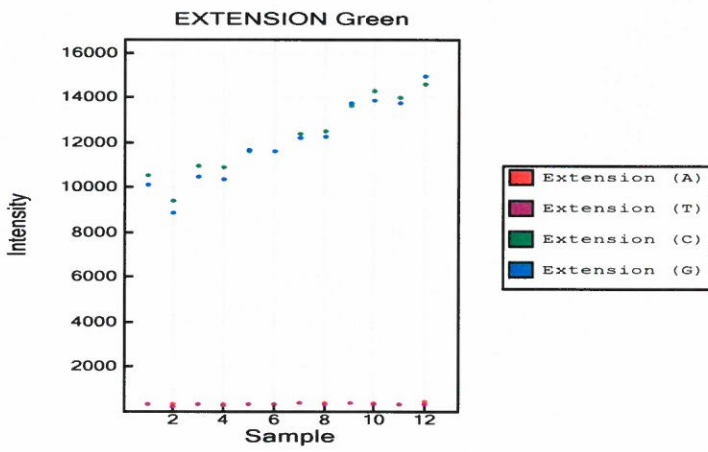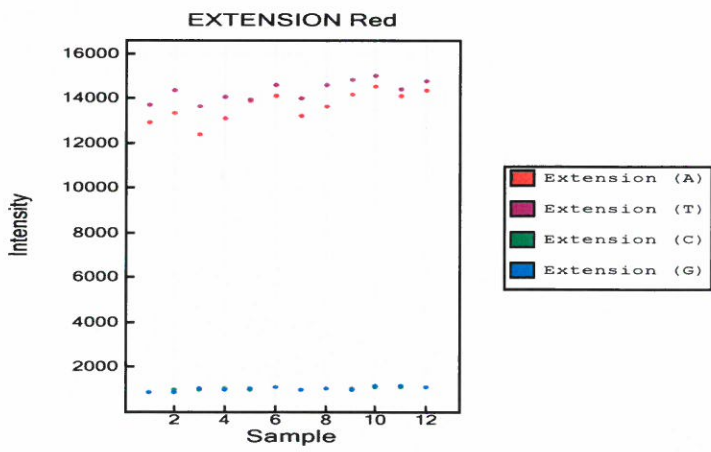

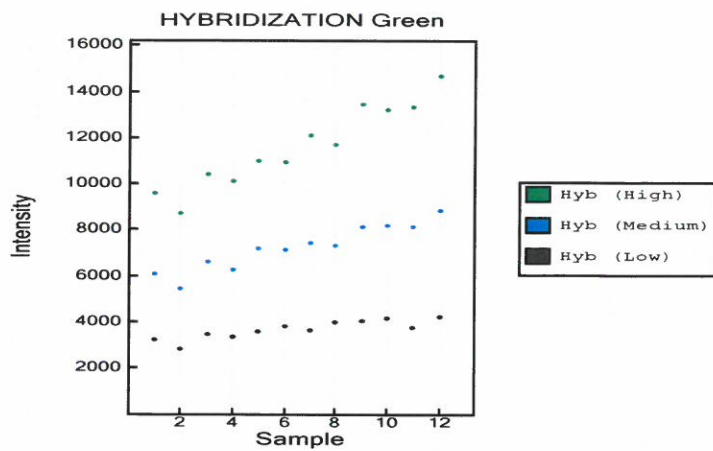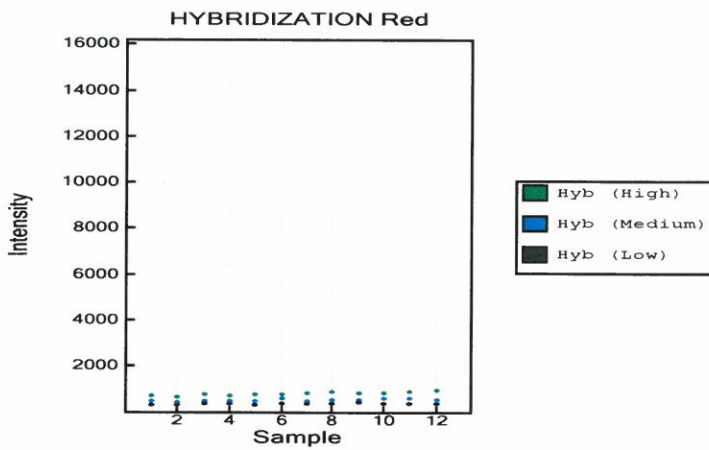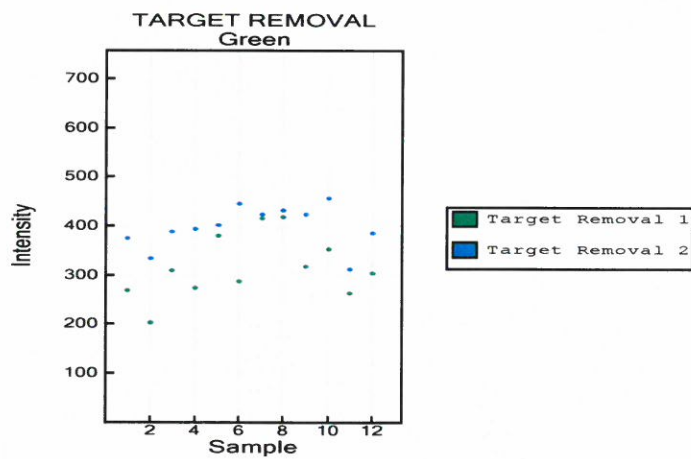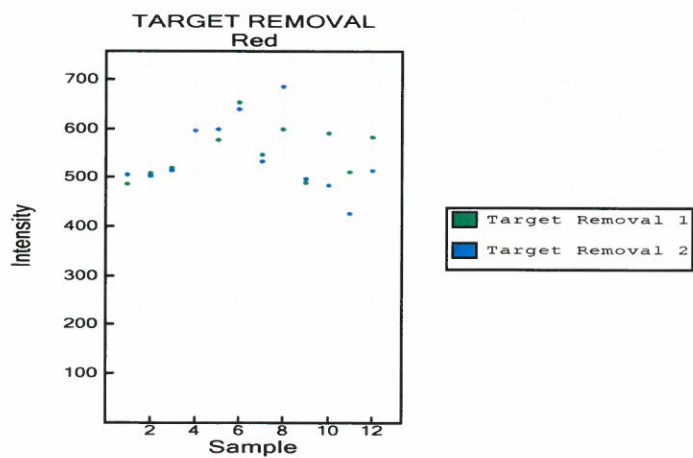

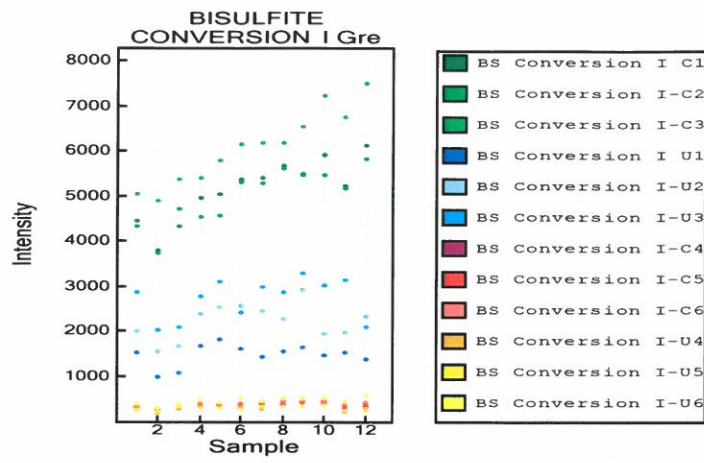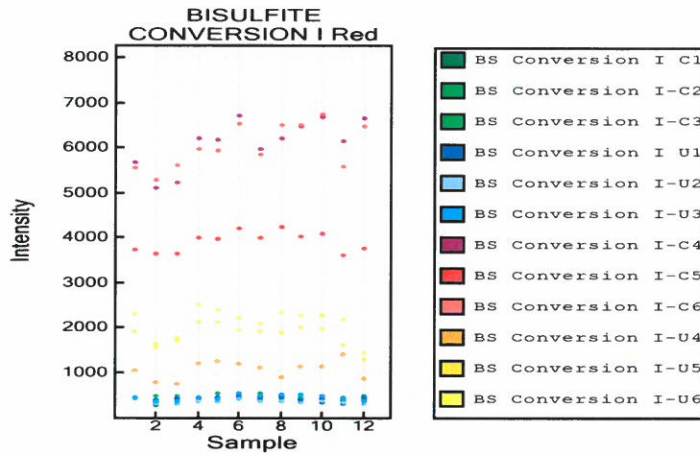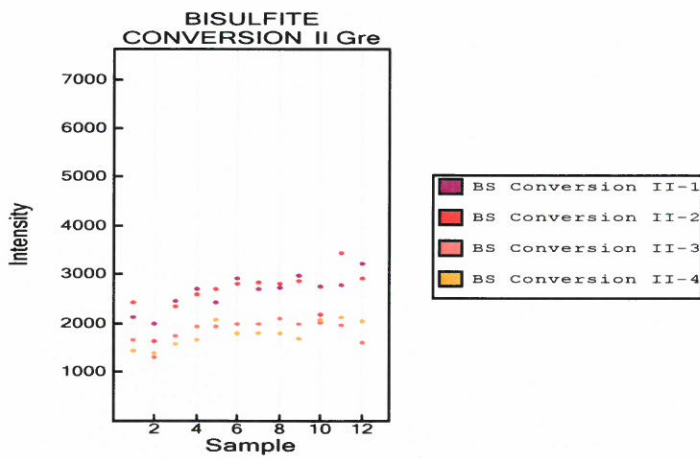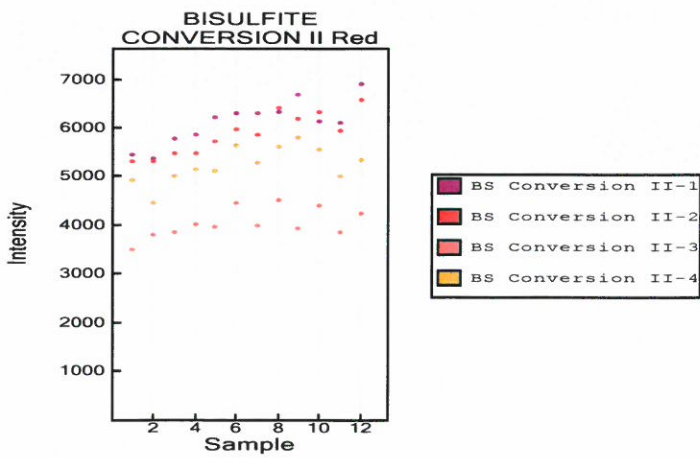

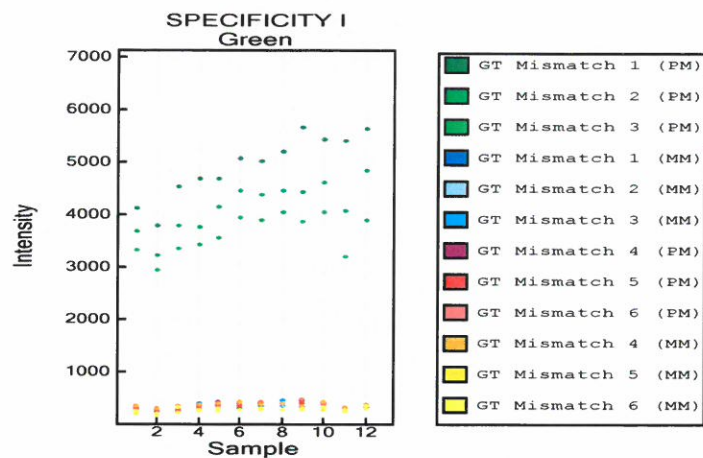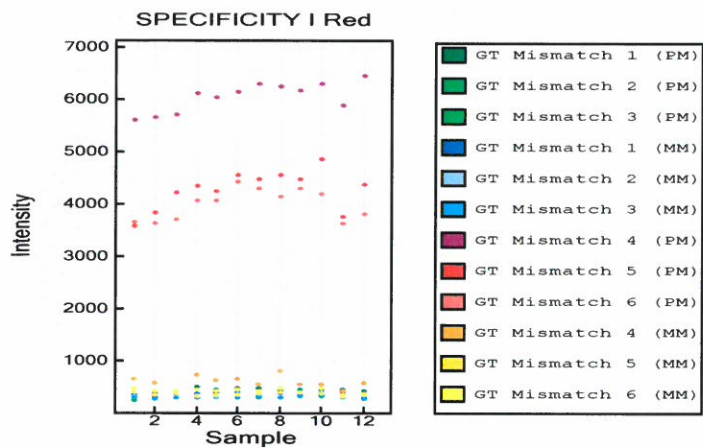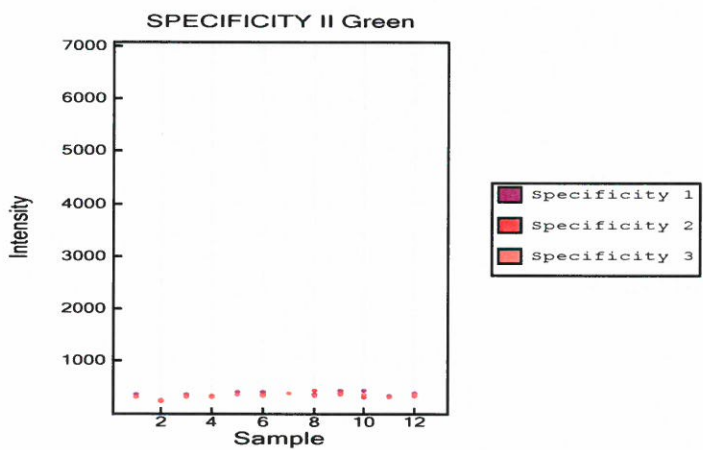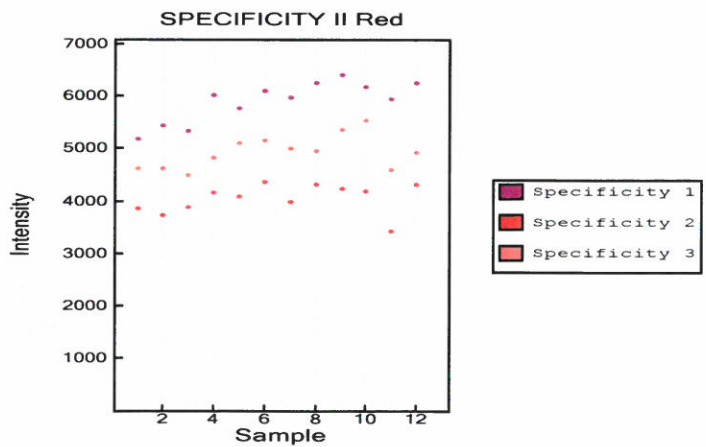

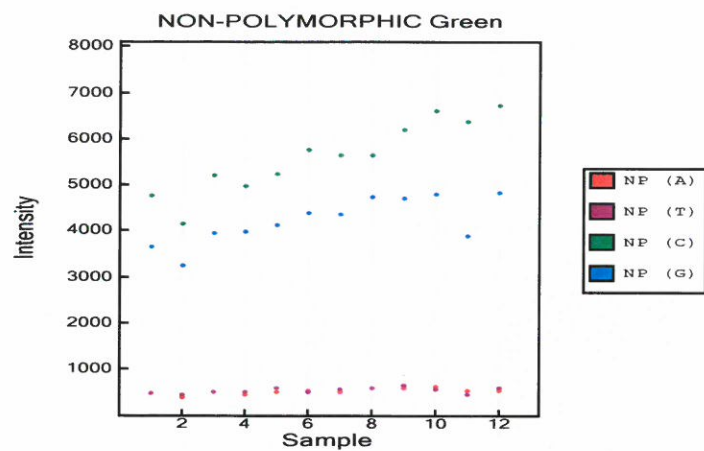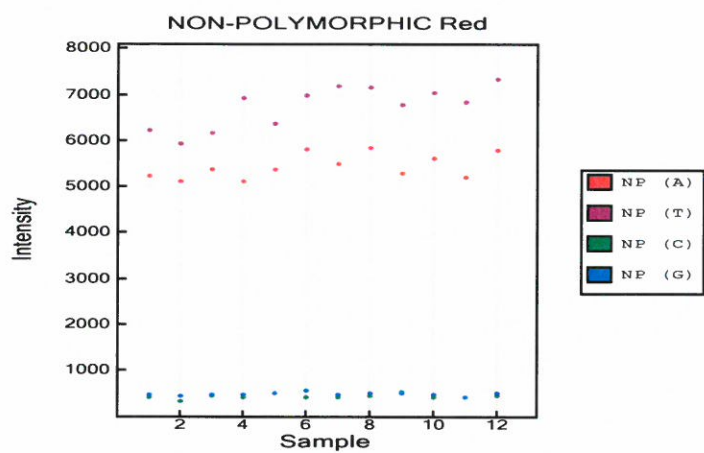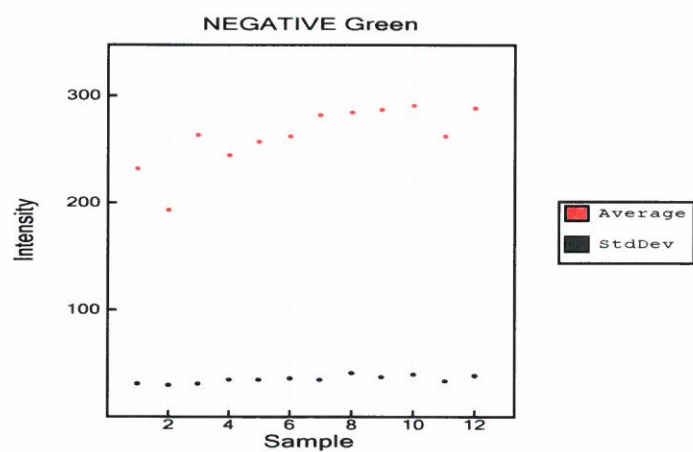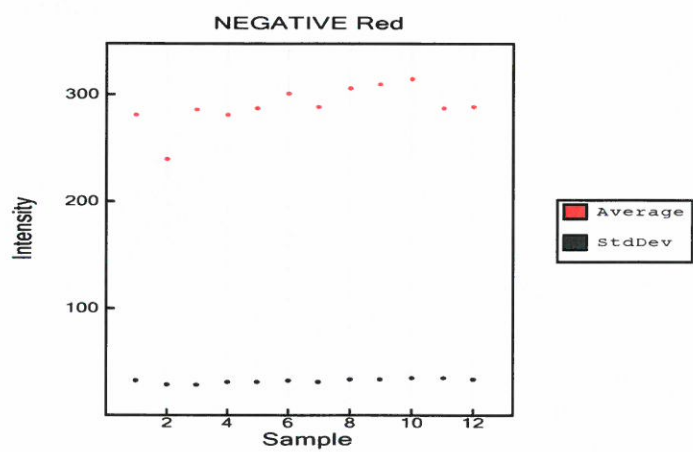

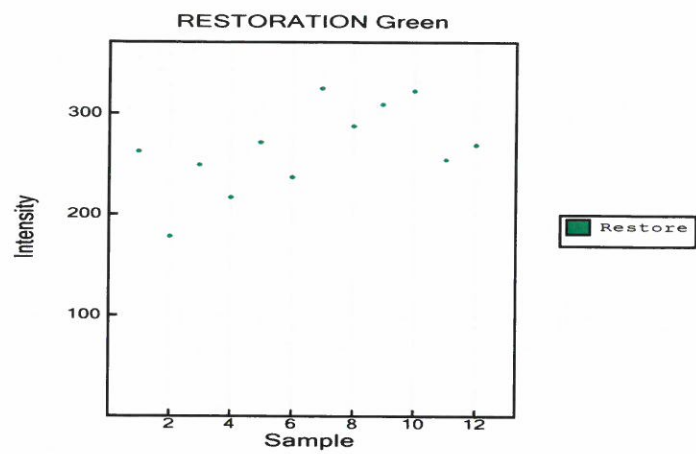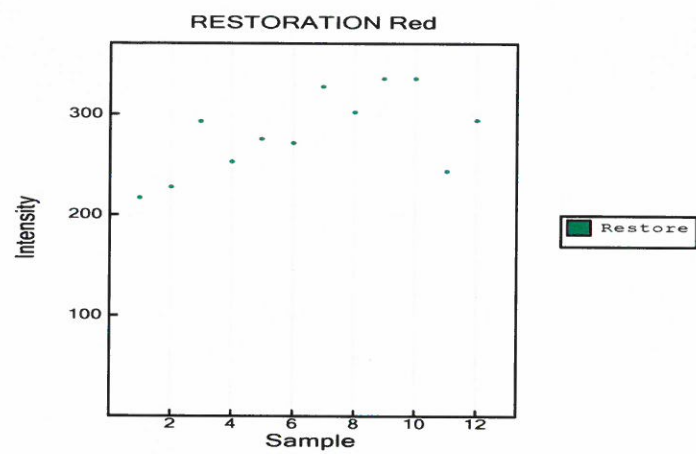

Supplement: S2 Fig — The Illumina Infinium HumanMethylation450 Beadchip arrays include several control probes for determining data quality, including sample-independent controls: staining controls, extension controls, target removal controls and hybridization controls. Sample-dependent controls: Bisulphite conversion I controls, Bisulphite conversion II controls, Specificity I controls, and Specificity II controls, Nonpolymorphic controls and Negative controls. Diagnostic plots of all control probes, visualized by illumines genome studios software, are presented for each of the 3 beadchip arrays. (ZIP) [file pone.0160319.s002.zip › Quality plots array 2.pdf]

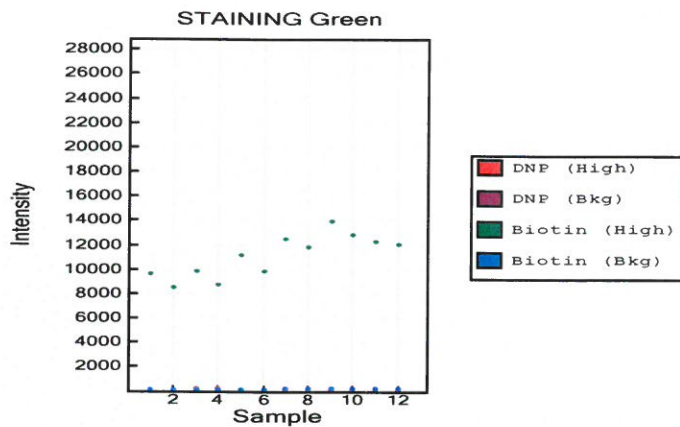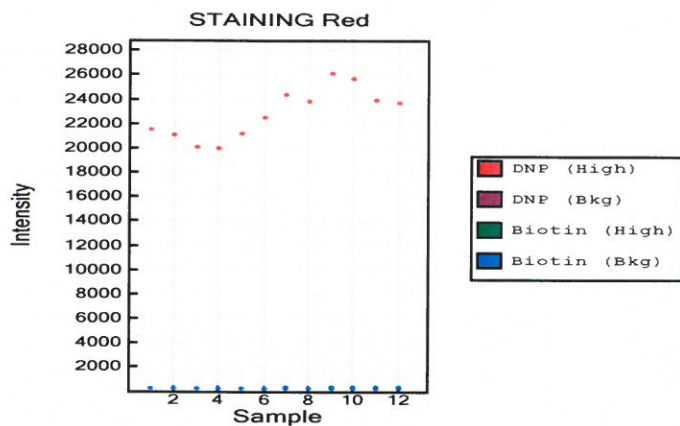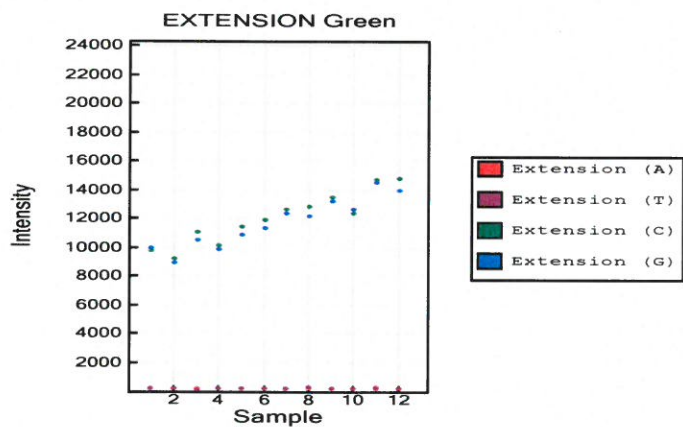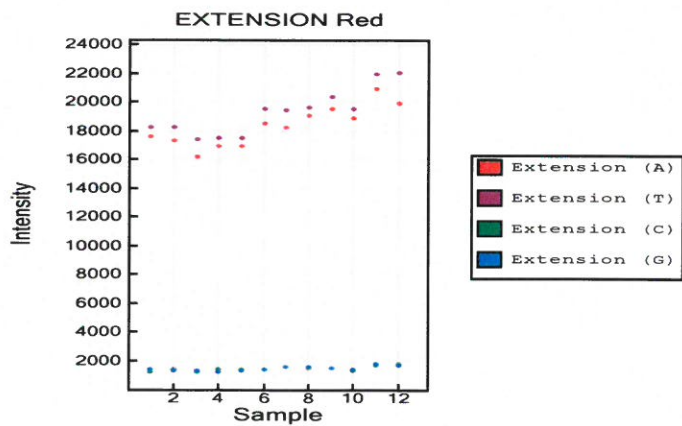

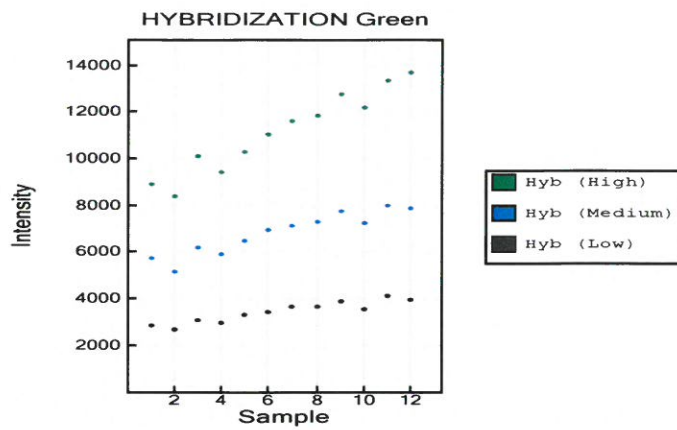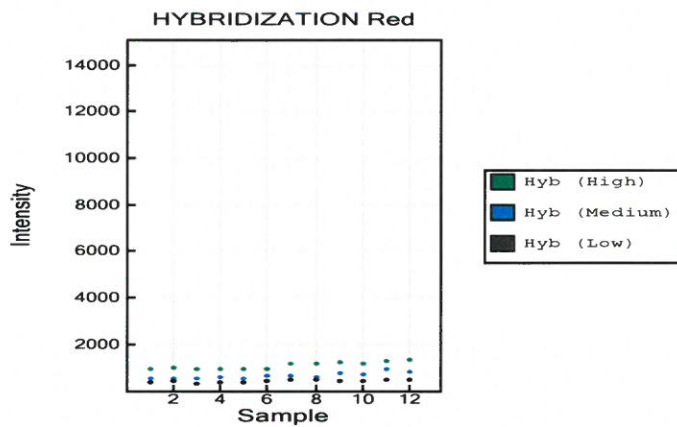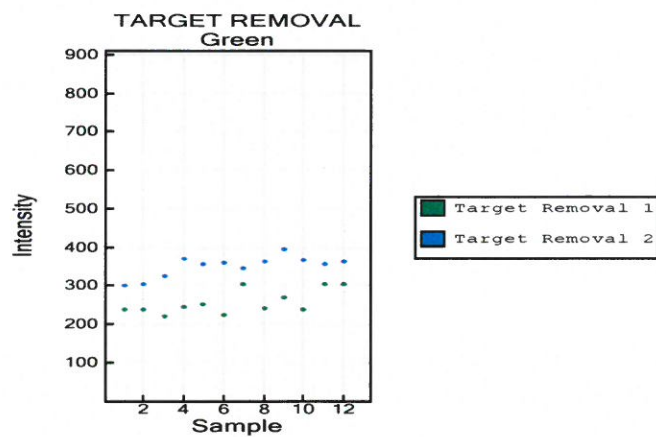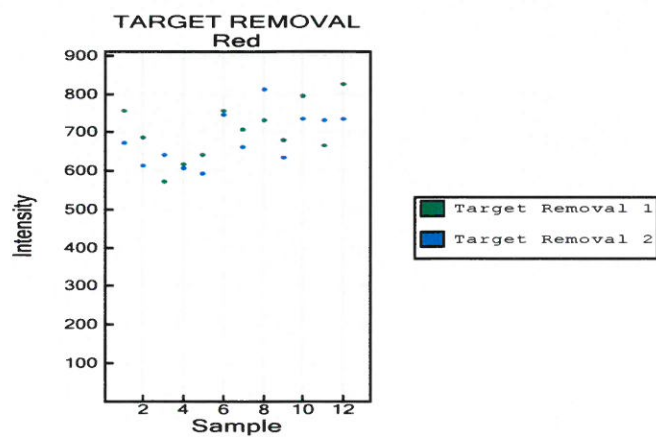

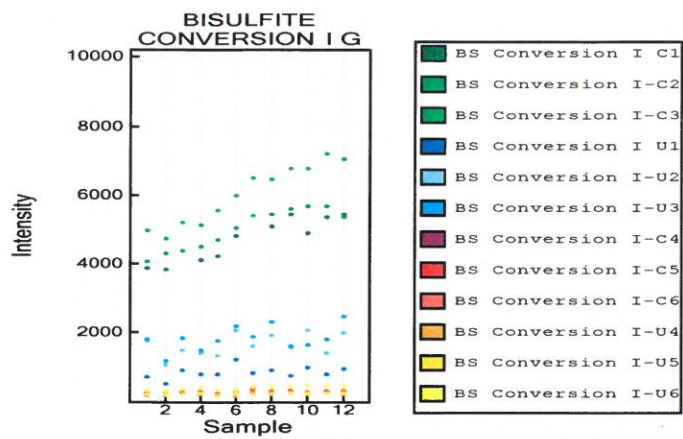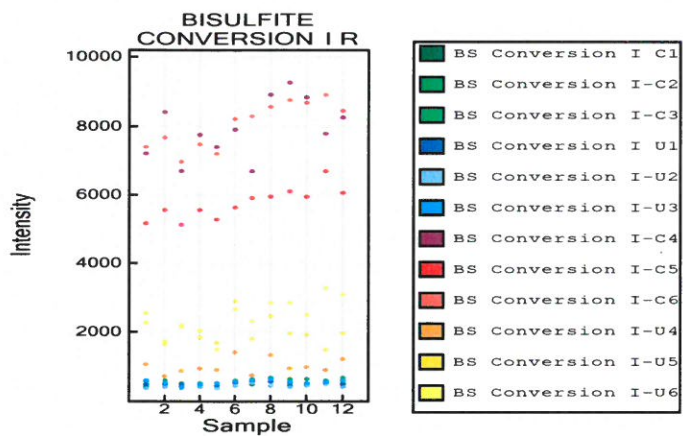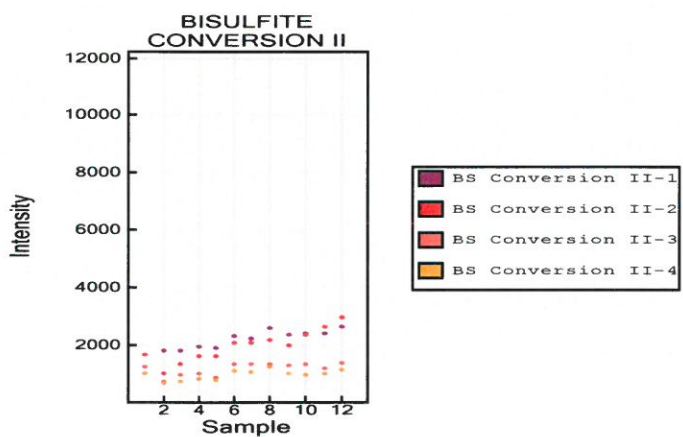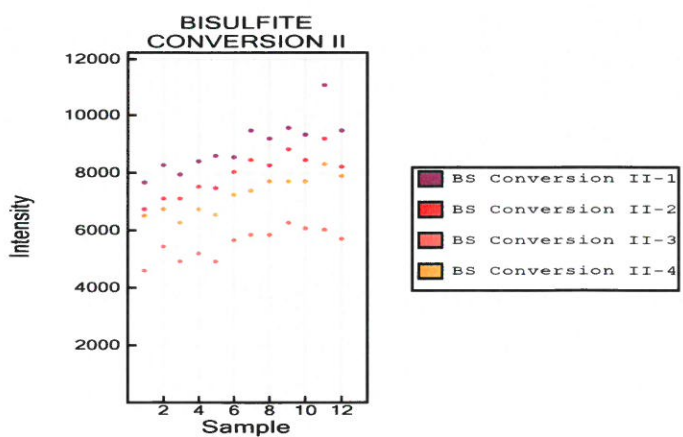

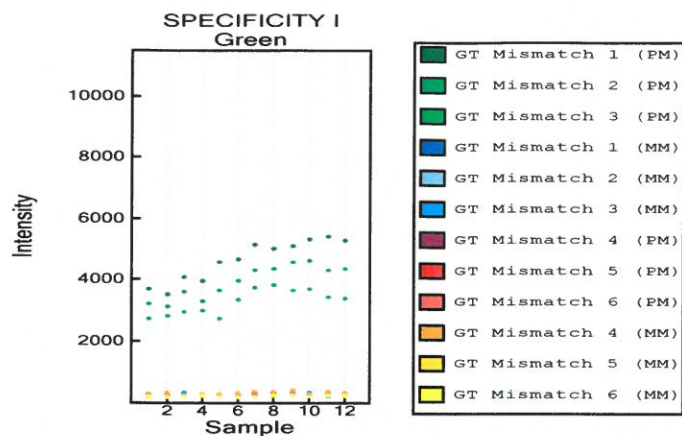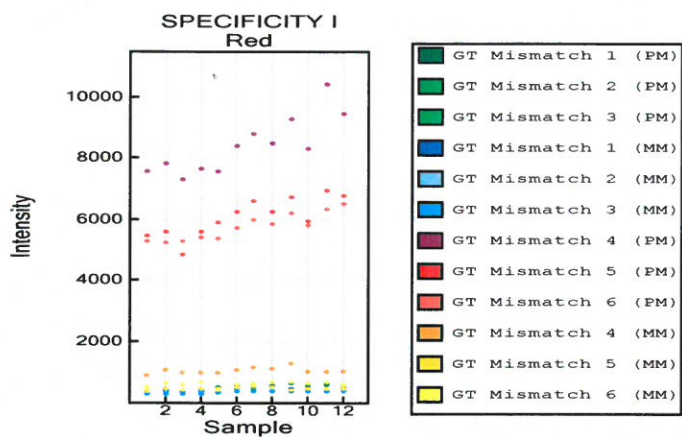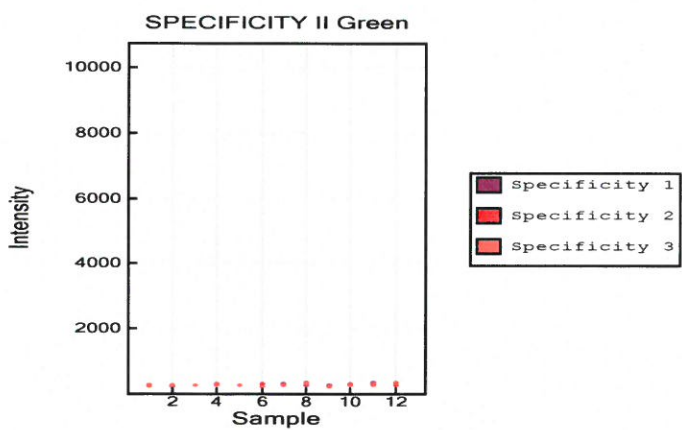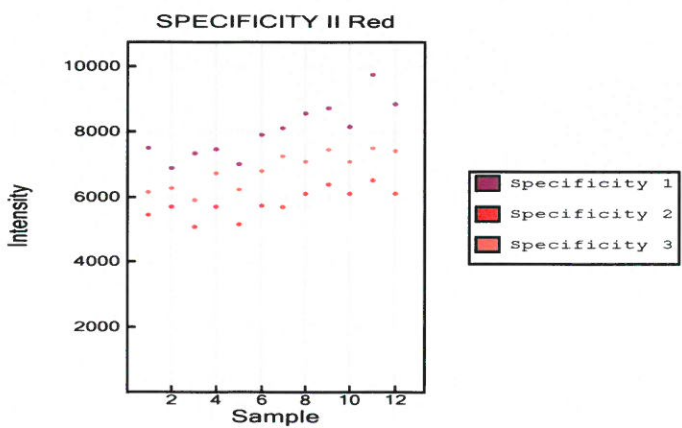

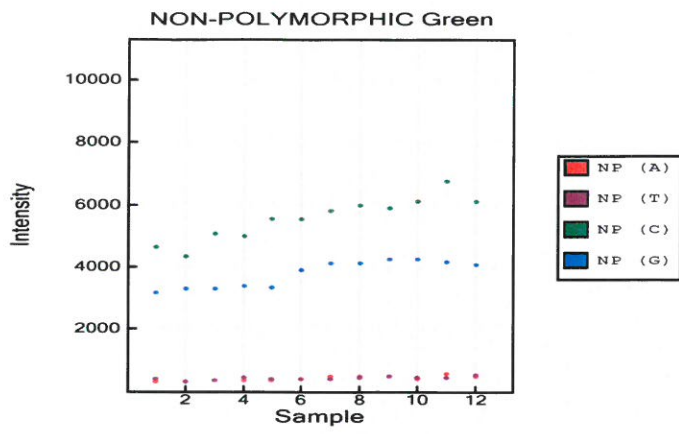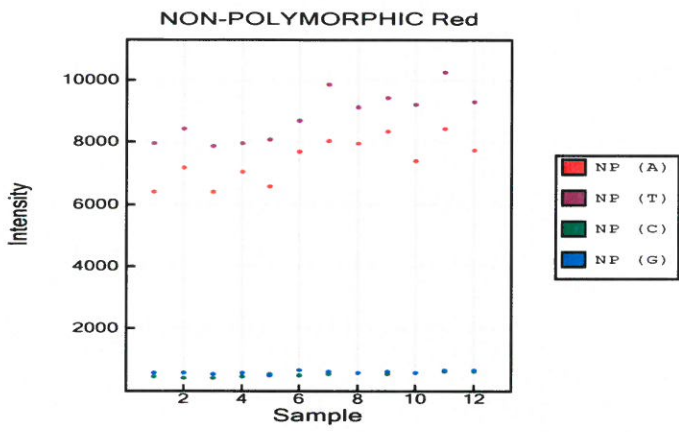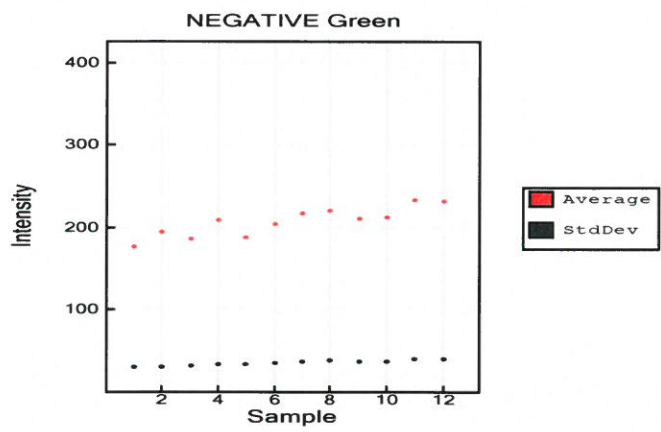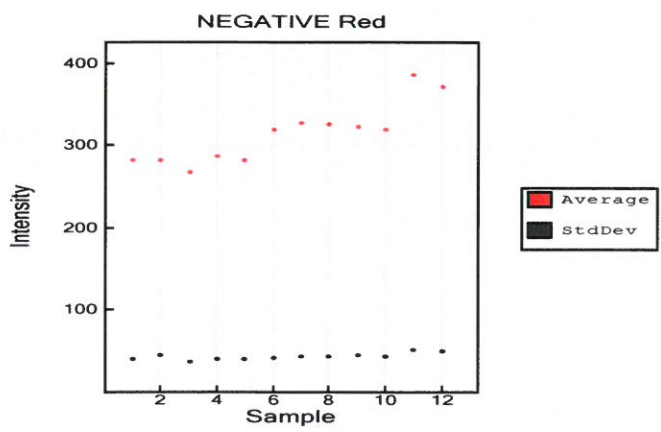

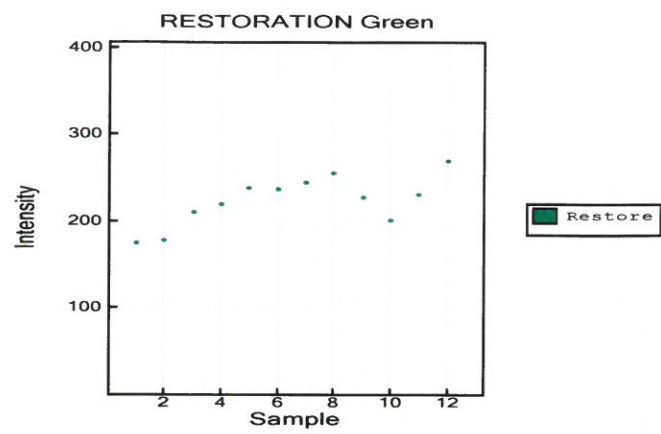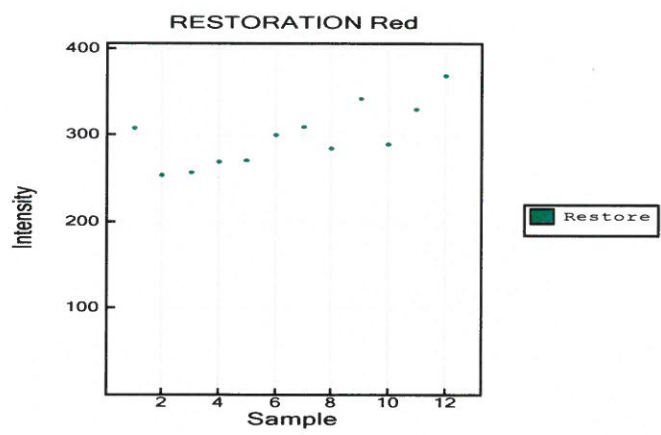

Supplement: S2 Fig — The Illumina Infinium HumanMethylation450 Beadchip arrays include several control probes for determining data quality, including sample-independent controls: staining controls, extension controls, target removal controls and hybridization controls. Sample-dependent controls: Bisulphite conversion I controls, Bisulphite conversion II controls, Specificity I controls, and Specificity II controls, Nonpolymorphic controls and Negative controls. Diagnostic plots of all control probes, visualized by illumines genome studios software, are presented for each of the 3 beadchip arrays. (ZIP) [file pone.0160319.s002.zip › Quality plots array 3.pdf]

Hierarchical clustering and heatmap of correlation coefficients

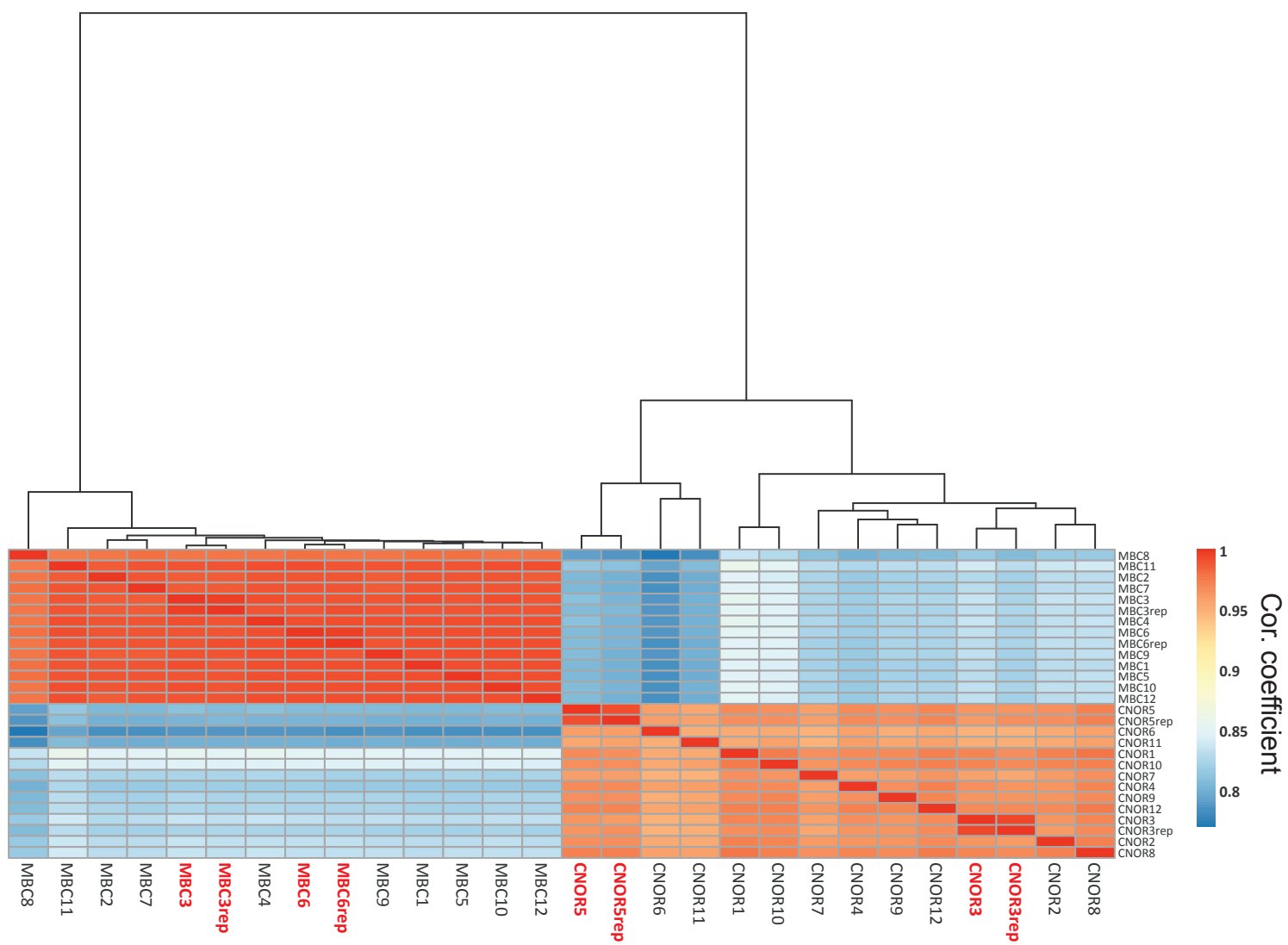

Supplement: S3 Fig — Four replicate samples, two maternal blood samples (MBC) and two CVS-samples, underwent independent bisulphite conversion and were analyzed on different beadchip arrays for the validation of DNA methylation data reproducibility. The figure shows hierarchical clustering and heatmap of correlation coefficients based on all 472K methylation sites. The four replicates MBC3, MBC6, CNOR3 and CNOR5 all shows the highest correlation between all samples. (PDF) [file pone.0160319.s003.pdf]

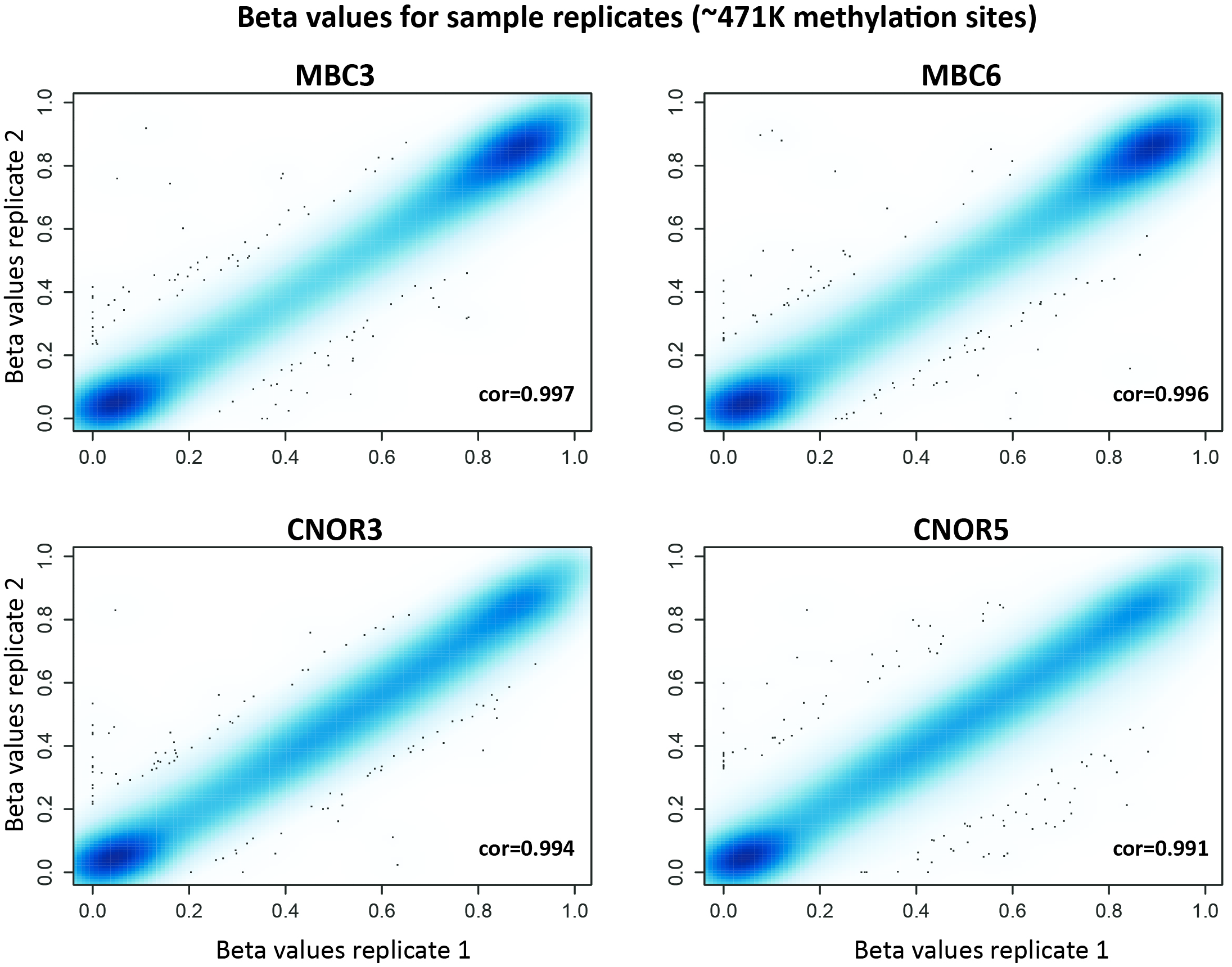

Supplement: S4 Fig — All four replicates have a correlation coefficient > 0.99 for all methylation sites. (JPG) [file pone.0160319.s004.jpg]
