# Supplementary material for: Microarray-Based Analysis of Methylation of 1st Trimester Trisomic Placentas from Down Syndrome, Edwards Syndrome and Patau Syndrome
Source: PLoS One. 2016 Aug 4;11(8):e0160319. doi: 10.1371/journal.pone.0160319 (PMC4973974; doi:10.1371/journal.pone.0160319)
Supplement: S5 Fig — The figure shows the mean delta β values for all unmethylated sites (average β < 0.2) for the four replicates. Since the replicates underwent independent bisulphite conversion, complete bisulphite conversion of unmethylated sites should results in reproducible low beta values. A. Delta β values for all unmethylated sites for the two maternal blood cell (MBC) replicate samples. Sorted with decreasing delta β values. X axis represents the numbers of unmethylated sites. B. The distribution of the 100 highest delta β values for the MBC samples. C. Delta β values for all unmethylated sites for the two CVS replicate samples (CNOR). D. The distribution of the 100 highest delta β values for the CVS samples. (PDF) [file pone.0160319.s005.pdf]

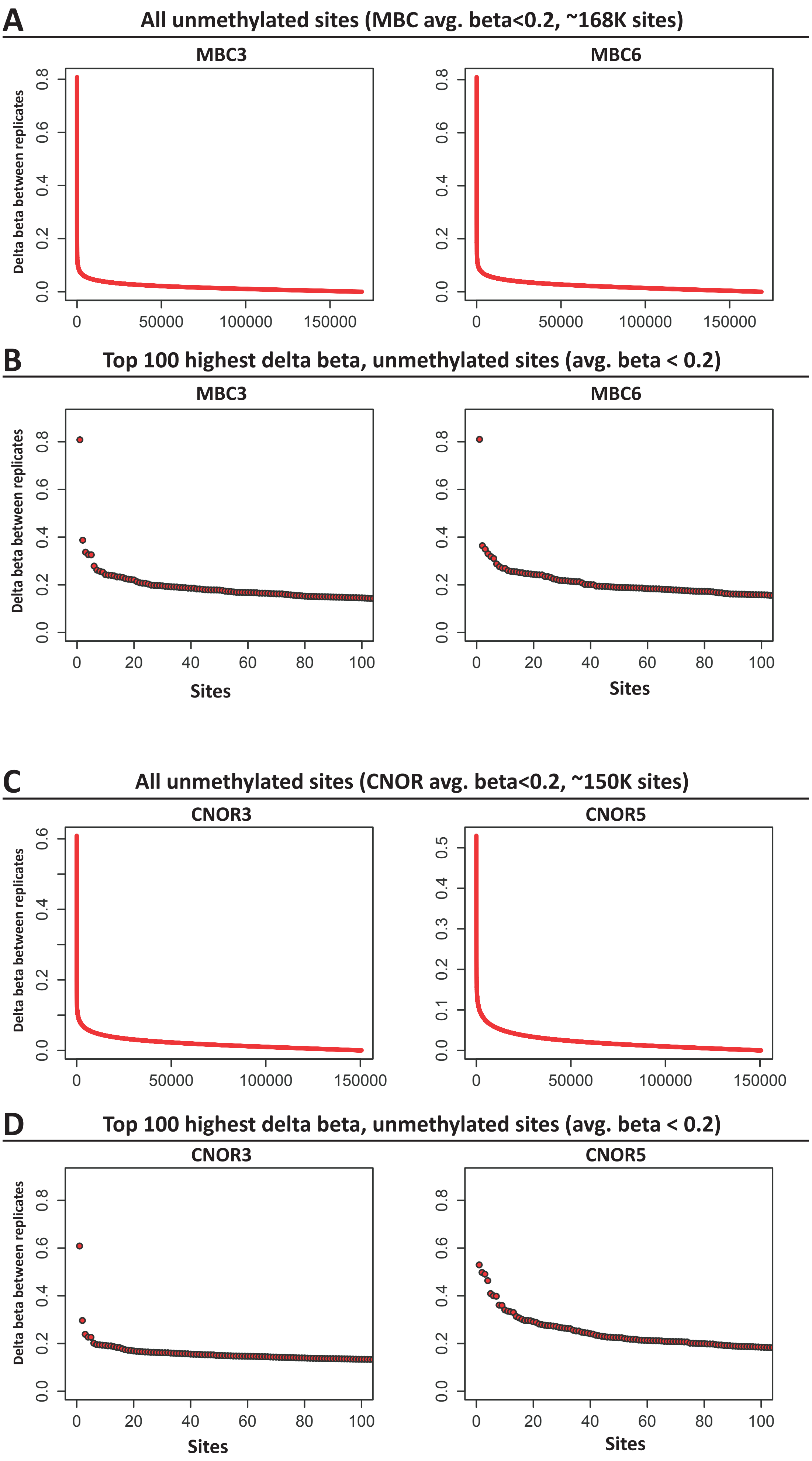
A
All unmethylated sites (MBC avg. beta<0.2, ~168K sites)
MBC3
MBC6
Delta beta between replicates
0.0
0.2
0.4
0.6
0.8
0
50000
100000
150000
B
Top 100 highest delta beta, unmethylated sites (avg. beta < 0.2)
MBC3
MBC6
Delta beta between replicates
0
20
40
60
80
100
Sites
C
All unmethylated sites (CNOR avg. beta<0.2, ~150K sites)
CNOR3
CNOR5
Delta beta between replicates
0.1
0.3
0.5
D
Top 100 highest delta beta, unmethylated sites (avg. beta < 0.2)
CNOR3
CNOR5
Delta beta between replicates
Sites
