# Supplementary material for: Microarray-Based Analysis of Methylation of 1st Trimester Trisomic Placentas from Down Syndrome, Edwards Syndrome and Patau Syndrome
Source: PLoS One. 2016 Aug 4;11(8):e0160319. doi: 10.1371/journal.pone.0160319 (PMC4973974; doi:10.1371/journal.pone.0160319)
Supplement: S1 Table — The table lists samples, fetal gender, year of sampling, sample material, chromosome analysis, gestational age (weeks), and maternal age (in years). (DOCX) [file pone.0160319.s007.docx]

| samples | Fetal Gender | Year of sampling | Sample material | Chromosome analysis | Gestational age (weeks) | Maternal age in years |
| --- | --- | --- | --- | --- | --- | --- |
| 1 | male | 2009 | Blood | Normal | 12 | 22 |
| 2 | male | 2012 | Blood | Normal | 13 | 34 |
| 3 | male | 2012 | Blood | Normal | 12 | 28 |
| 4 | male | 2011 | Blood | Normal | 12 | 26 |
| 5 | male | 2011 | Blood | Normal | 12 | 31 |
| 6 | female | 2009 | Blood | Normal | 12 | 35 |
| 7 | female | 2012 | Blood | Normal | 11 | 25 |
| 8 | female | 2010 | Blood | Normal | 13 | 28 |
| 9 | female | 2011 | Blood | Normal | 12 | 23 |
| 10 | female | 2010 | Blood | Normal | 13 | 37 |
| 1 | male | 2012 | CVS | Normal | 12+2 | 33 |
| 2 | male | 2012 | CVS | Normal | 13+0 | 32 |
| 3 | male | 2011 | CVS | Normal | 13+3 | 39 |
| 4 | male | 2011 | CVS | Normal | 13+2 | 39 |
| 5 | male | 2010 | CVS | Normal | 14+5 | 34 |
| 6 | male | 2010 | CVS | Normal | 13+5 | 38 |
| 7 | female | 2012 | CVS | Normal | 13+6 | 33 |
| 8 | female | 2012 | CVS | Normal | 13+0 | 34 |
| 9 | female | 2011 | CVS | Normal | NA | 32 |
| 10 | female | 2011 | CVS | Normal | 12+5 | 34 |
| 11 | female | 2010 | CVS | Normal | 14+0 | 41 |
| 12 | female | 2010 | CVS | Normal | 13+4 | 37 |
| 1 | male | 2011 | CVS | Trisomy 21 | 14+0 | 31 |
| 2 | male | 2011 | CVS | Trisomy 21 | 13+5 | 38 |
| 3 | male | 2011 | CVS | Trisomy 21 | 13+2 | 38 |
| 4 | male | 2011 | CVS | Trisomy 21 | 14+0 | 41 |
| 5 | male | 2011 | CVS | Trisomy 21 | 12+0 | 36 |
| 6 | male | 2011 | CVS | Trisomy 21 | 13+3 | 41 |
| 7 | female | 2011 | CVS | Trisomy 21 | 12+6 | 38 |
| 8 | female | 2011 | CVS | Trisomy 21 | 12+3 | 38 |
| 9 | female | 2011 | CVS | Trisomy 21 | 15+0 | 17 |
| 10 | female | 2011 | CVS | Trisomy 21 | 14+5 | 40 |
| 11 | female | 2011 | CVS | Trisomy 21 | 13+0 | 42 |
| 12 | female | 2011 | CVS | Trisomy 21 | 14+0 | 36 |
| 1 | male | 2011 | CVS | Trisomy 18 | 12+1 | 45 |
| 2 | male | 2011 | CVS | Trisomy 18 | 12+0 | 41 |
| 3 | male | 2012 | CVS | Trisomy 18 | 13+0 | 41 |
| 4 | male | 2010 | CVS | Trisomy 18 | 12+6 | 32 |
| 5 | male | 2009 | CVS | Trisomy 18 | 12+1 | 31 |
| 6 | male | 2008 | CVS | Trisomy 18 | 13+0 | 32 |
| 7 | female | 2007 | CVS | Trisomy 18 | 11+0 | 27 |
| 8 | female | 2012 | CVS | Trisomy 18 | 12+0 | 44 |
| 9 | female | 2012 | CVS | Trisomy 18 | 12+0 | 32 |
| 10 | female | 2009 | CVS | Trisomy 18 | 12+5 | 36 |
| 11 | female | 2009 | CVS | Trisomy 18 | 11+6 | 31 |
| 12 | female | 2009 | CVS | Trisomy 18 | 14+0 | 36 |
| 1 | male | 2009 | CVS | Trisomy 13 | 13+2 | 33 |
| 2 | male | 2009 | CVS | Trisomy 13 | 13+6 | 38 |
| 3 | male | 2009 | CVS | Trisomy 13 | 13+2 | 30 |
| 4 | male | 2010 | CVS | Trisomy 13 | 12+4 | 28 |
| 5 | female | 2008 | CVS | Trisomy 13 | 12+6 | 29 |
| 6 | female | 2011 | CVS | Trisomy 13 | 13+0 | 25 |
