# Supplementary material for: Microarray-Based Analysis of Methylation of 1st Trimester Trisomic Placentas from Down Syndrome, Edwards Syndrome and Patau Syndrome
Source: PLoS One. 2016 Aug 4;11(8):e0160319. doi: 10.1371/journal.pone.0160319 (PMC4973974; doi:10.1371/journal.pone.0160319)
Supplement: S3 Table — Numbers (and percentages) of sites with increasing delta β value thresholds. A All methylation sites. B Unmethylated Sites (average delta β < 0.2). (PDF) [file pone.0160319.s009.pdf]

Overview of methylation differences for sample replicates

A

All methylation sites

Sample replicates	# sites Δβ > 0.1	# sites Δβ > 0.2	# sites Δβ > 0.3	# sites Δβ > 0.4
MBC3	7,696 (1.635%)	253 (0.054%)	32	8
MBC6	11,573 (2.458%)	304 (0.066%)	32	11
CNOR3	15,616 (3.317%)	307 (0.065%)	22	10
CNOR5	19,347 (4.11%)	1,171 (0.249%)	165	36

B

Unmethylated sites (avg. beta < 0.2 for MBC or CNOR sample groups)

Sample replicates	# sites Δβ > 0.1	# sites Δβ > 0.2	# sites Δβ > 0.3	# sites Δβ > 0.4
MBC3	498 (0.295%)	25 (0.015%)	5	1
MBC6	760 (0.45%)	41 (0.024%)	6	1
CNOR3	718 (0.437%)	12 (0.007%)	3	1
CNOR5	1,820 (1.108%)	106 (0.065%)	19	6
